# Rates of maternal weight gain over the course of pregnancy and offspring risk of neurodevelopmental disorders

**DOI:** 10.1186/s12916-023-02799-6

**Published:** 2023-03-23

**Authors:** Shuyun Chen, Mengyu Fan, Brian K. Lee, Christina Dalman, Håkan Karlsson, Renee M. Gardner

**Affiliations:** 1 Department of Global Public Health, Karolinska Institutet, Stockholm, Sweden; 2 Department of Epidemiology and Biostatistics, Drexel University School of Public Health, Philadelphia, PA USA; 3 A.J. Drexel Autism Institute, Philadelphia, PA USA; 4 Centre for Epidemiology and Community Medicine, Stockholm County Council, Stockholm, Sweden; 5 Department of Neuroscience, Karolinska Institutet, Stockholm, Sweden

**Keywords:** Gestational weight gain, Autism spectrum disorder (ASD), Intellectual disability (ID), Attention deficit/hyperactivity disorder (ADHD)

## Abstract

**Background:**

Previous studies have suggested that gestational weight gain (GWG) outside an optimal range increases the risks of neurodevelopmental disorders (NDDs) in offspring including autism spectrum disorder (ASD), intellectual disability (ID), and attention deficit/hyperactivity disorder (ADHD). The sequential development of the fetal brain suggests that its vulnerability may vary depending on the timing of exposure. Therefore, we aimed to investigate the associations of not only gestational age-standardized total GWG (GWG *z*-scores) but also the rate of GWG (RGWG) in the second and third trimesters with risks of NDDs in offspring.

**Methods:**

In this population-based cohort study, we used maternal weight data from antenatal care records collected for 57,822 children born to 53,516 mothers between 2007 and 2010 in the Stockholm Youth Cohort. Children were followed from 2 years of age to December 31, 2016. GWG *z*-scores and RGWG (kg/week) in the second and third trimesters were considered as continuous variables in cox regression models, clustered on maternal identification numbers. Nonlinear relationships were accommodated using restricted cubic splines with 3 knots. RGWG were also categorized according to the 2009 US Institute of Medicine (IOM) guidelines for optimal GWG. According to the IOM guidelines, the optimal rate of GWG for the second and third trimesters for underweight, normal weight, overweight, and obese categories were 0.44–0.58, 0.35–0.50, 0.23–0.33, and 0.17–0.27 kg/week, respectively.

**Results:**

During a mean follow-up of 5.4 years (until children were on average 7.4 years old), 2205 (3.8%) children were diagnosed with NDDs, of which 1119 (1.9%) received a diagnosis of ASD, 1353 (2.3%) ADHD, and 270 (0.5%) ID. We observed a J-shaped association between total GWG *z*-score and offspring risk of NDDs, with higher total GWG (GWG *z*-score = 2) associated with 19% increased risk of any NDD (95% CI = 3–37%) and lower total GWG (GWG *z*-score = − 2) associated with 12% increased risk of any NDDs (95% CI = 2–23%), compared to the reference (GWG *z*-score = 0). In the second trimester, lower RGWG (0.25 kg/week) was associated with a 9% increased risk of any NDD diagnosis (95% CI = 4–15%) compared to the median of 0.57 kg/week, with no apparent relationship between higher RGWG and risk of NDDs. In the third trimester, there was no apparent association between lower RGWG and risk of NDDs, though higher RGWG (1 kg/week) was associated with a 28% increased risk of NDD diagnosis (95% CI = 16–40%), compared to the median (0.51 kg/week). When considering categorized RGWG, we found that slow weight gain in the second trimester followed by rapid weight gain in the third trimester most significantly increased the risk of ADHD (HR_adjusted_ = 1.55, 1.13–2.13) and ID (HR_adjusted_ = 2.53, 1.15–5.55) in offspring. The main limitations of our study are the relatively few years for which detailed GWG data were available and the relatively short follow-up for the outcomes, limiting power to detect associations and misclassifying children who receive an NDD diagnosis later in childhood.

**Conclusions:**

The relationship between maternal weight gain and children’s risk of NDDs varied according to timing in pregnancy, with the greatest risks associated with slow weight gain in the second trimester and rapid weight gain in the third trimester.

**Supplementary Information:**

The online version contains supplementary material available at 10.1186/s12916-023-02799-6.

## Background

Autism spectrum disorder (ASD), Intellectual disability (ID), and attention deficit/hyperactivity disorder (ADHD) are three common neurodevelopmental disorders (NDDs) in children that often co-occur [1–4]. Their relatively high prevalence and the often life-long need for social support in affected individuals can place great burdens on their families and society as a whole [4]. Although highly heritable and linked to both rare inherited and de novo mutations, their underlying etiologies do not appear to be completely explained by genetics, indicating contributions also from other biological, environmental, and social factors [1–4].

Though the main intention of the Institute of Medicine (IOM) guideline for optimal gestational weight gain is to provide clinicians with a basis for practice [5], evidence has emerged in the past decades for an association between maternal total GWG outside of the optimal range defined by the guidelines and children’s risk of NDDs, such as ASD [6, 7], ID [8], and ADHD [9]. One limitation of previous studies using total GWG was that they did not take length of pregnancy into consideration, which made it difficult to disentangle the effects of GWG on adverse NDD outcomes from the effects of the gestational duration [10]. There is a growing appreciation for using the trimester-specific rate of weight gain and *z*-score charts of maternal weight-gain-for-gestational-age as a measure of pregnancy weight gain [10–12].

The rapid growth of fetal brains makes them particularly vulnerable to damage by nutritional and metabolic disturbances compared to adult brains [13]. The sequential growth and development of structural and functional components of the fetal brain is a dynamic process [14], and the vulnerability of the fetal brain varies across specific periods of exposure to environmental stressors [13]. However, the effects of abnormal rates of GWG (RGWG) during specific gestational periods, especially in the second and third trimesters when most weight gain occurs [15], on the risk of NDDs in offspring remain unclear, as previous studies lacked longitudinal measures of maternal weight and relied only on total GWG.

In this Swedish population-based cohort study, we aimed to investigate the relationships of both Swedish gestational age-standardized total GWG *z*-scores and rate of GWG in the second and third trimesters, with risks of NDDs (i.e., ASD, ID, and ADHD) in offspring.

## Methods

### Study population

We used data from the Stockholm antenatal care record system (Obstetrix) [11, 16] from January 1, 2007, to December 31, 2010, which was linked to the Medical Birth Register (MBR) and nested within the Stockholm Youth Cohort (SYC). Details of the SYC design have been described elsewhere [17, 18]. Information concerning exposures, outcomes, and covariates was extracted from national and regional health registers and administrative registers. Ethics approval was obtained from the Stockholm regional ethical review committee (DNR 2010/1185-31/5, 2016/987-32). Informed consent was not required for the analysis of anonymized register data.

We included all children born from January 1, 2007, to December 31, 2010, in Stockholm and with maternal weight measurements throughout pregnancy. All children were followed up from 2 years of age until December 31, 2016, or the date of NDD diagnosis, emigration, or death, whichever came first. We excluded children from multiple births or without maternal height information and further excluded children whose mothers did not have at least one weight recorded within each trimester (14 and 28 weeks as trimester cut-points). Children who received a diagnosis of an NDD or who emigrated or died before their second birthday were also excluded (Additional file 1: Fig. S1A). Our final study sample included 57,822 children born to 53,516 mothers. Excluded children had a slightly higher risk of ID diagnosis and were more likely to be born to migrant parents and low-income families (Additional file 1: Table S1).

### Case ascertainment

Cases of ASD, ADHD, and ID were ascertained using information gathered from all potential care pathways in Stockholm County (Additional file 1: Table S2) [17–19]. Briefly, the International Classification of Diseases, 10th revision (ICD-10; F84 for ASD, F90 for ADHD, and F70–F79 for ID) and additional information from the Prescription Drug Register (methylphenidate or atomoxetine for ADHD definition) were used to define the diagnostic groups. Our primary analysis considered any NDD diagnosis as an outcome, along with any diagnosis of ASD, ADHD, or ID, though individuals can be included in more than one outcome category (e.g., those diagnosed with “ASD with ID” would be included in both the ASD and ID outcomes). In secondary analyses, we considered mutually exclusive outcomes defined as follows: ASD only (no ADHD or ID), ADHD only (no ASD or ID), ASD with ADHD (no ID), ASD with ID (not excluding ADHD), and ID without ASD (no ASD, not excluding ADHD) (Additional file 1: Fig. S1B).

### Exposures: GWG and RGWG in different pregnancy stages

The Obstetrix record system contains maternal weight data measured by midwives during each antenatal visit throughout pregnancy, beginning in 2006. Weight observations < 30 kg or > 200 kg were censored, as values indicating a weekly weight gain or weight loss > 5 kg. A total of 318,487 serial maternal weight measurements from 57,822 pregnancies were included in the final sample. The number of weight measurements per pregnancy differed, with a median of 5 [interquartile range (IQR): 4–7]. The frequency of measurements increased over time in pregnancy, with a median of 1 (IQR: 1–1) in the first, 1 (IQR: 1–2) in the second, and 2 (IQR: 1–4) in the third trimesters.

The rate of weight gain (kg/week) during the second trimester (RGWG-T2) was calculated using the difference in the last weight measurement in the second trimester and the last weight measurement taken in the first trimester divided by the gestational week interval between the measurements. As the weight gain in the first trimester was usually small (i.e., ~ 1–2 kg) compared to the second and third trimesters [10, 20] and most women only had one measurement in the first trimester, we recoded the timing of measurement in the first trimester as 13 wkGA if the measurement was taken before 13 wkGA to avoid underestimating RGWG in the second trimester. The rate of weight gain (kg/week) during the third trimester (RGWG-T3) was similarly calculated, using the difference in the final weight measurement before delivery and the last weight measurement taken in the second trimester divided by the gestational week interval between the measurements.

Given that the total GWG (kg) is influenced by gestational duration, which is also associated with the risk of NDDs, we standardized the total GWG to *z*-scores according to Swedish standards [11] taking gestational week of birth into account. For comparison to the *z*-score analysis, total GWG in kilograms was calculated as the difference in maternal weight between the first antenatal visit (median 9.4, IQR: 8.1–10.7 weeks) and the last antenatal visit (median: 37.1, IQR: 36.0–38.3 weeks).

While our primary analysis relied on the continuous measures described above, we also created categories for “optimal,” “insufficient,” or “excessive” rates of weight gain in the second and third trimesters based on IOM recommendations for each BMI category [20] (optimal ranges for underweight 0.44–0.58 kg/week; normal BMI 0.35–0.50 kg/week; overweight 0.23–0.33 kg/week; obese 0.17–0.27 kg/week). We hypothesized a U-shaped association between RGWG and offspring risk of NDDs; values furthest from the optimal range may therefore represent the highest risk categories. Following from previous work [21], we further divided the “insufficient” and “excessive” categories at their respective medians (by BMI category) to create extended rate categories: “optimal,” “extremely insufficient,” “insufficient,” “excessive,” and “extremely excessive.” Slow or fast weight gain in the second trimester may induce either catch-up or reduced weight gain in the third trimester due to effective gestational weight management. Taking RGWG-T2 and RGWG-T3 together, we generated the following groups: (1) optimal at both time points (optimal/optimal, reference), (2) optimal/insufficient, (3) optimal/excessive, (4) insufficient/optimal, (5) insufficient/insufficient, (6) insufficient/ excessive, (7) excessive/optimal, (8) excessive/insufficient, and (9) excessive/excessive. Finally, three total GWG categories were defined for each BMI category: “optimal,” “insufficient,” or “excessive” (optimal ranges for underweight 12.5–18 kg; normal BMI 11.5–16 kg; overweight 7–11.5 kg; obese 5–9 kg) [20].

### Covariates

Maternal weight at the first antenatal visit was used to approximate baseline maternal BMI (in kg/m^2^), at a median of 9.4 (IQR: 8.1–10.7) weeks and was categorized as underweight (BMI < 18.5), normal BMI (18.5 ≤ BMI < 25), overweight (25 ≤ BMI < 30), or obese (BMI ≥ 30). The following covariates were considered as potential confounders and included in the study: child’s sex, birth year, household income quintiles at birth, maternal age at birth, maternal education level, parental birth region (i.e., maternal and paternal region of birth), interpregnancy interval (IPI), maternal smoking during pregnancy, and maternal psychiatric history prior to the birth of the child, parameterized as specified in Table 1. A directed acyclic graph describing the associations between covariates, exposures, and outcomes is presented in Additional file 1: Fig. S2.

**Table 1:** Characteristics of the study cohort by rate of gestational weight gain category

Characteristic	RGWG-T2			RGWG-T3		
	Optimal	Insufficient	Excessive	Optimal	Insufficient	Excessive
***N***	10,480	7647	39,695	13,526	10,163	34,133
**Weight measurements, median (IQR)**						
1st trimester	1.0 (1.0, 1.0)	1.0 (1.0, 1.0)	1.0 (1.0, 1.0)	1.0 (1.0, 1.0)	1.0 (1.0, 1.0)	1.0 (1.0, 1.0)
2nd trimester	1.0 (1.0, 2.0)	1.0 (1.0, 2.0)	1.0 (1.0, 2.0)	1.0 (1.0, 2.0)	1.0 (1.0, 2.0)	1.0 (1.0, 2.0)
3rd trimester	2.0 (1.0, 4.0)	3.0 (1.0, 4.0)	2.0 (1.0, 4.0)	2.0 (1.0, 4.0)	2.0 (1.0, 4.0)	2.0 (1.0, 4.0)
**Gestational week at the first weight measurement, median (IQR)**						
1st trimester	9.4 (8.1, 10.7)	9.3 (8.0, 10.6)	9.1 (7.9, 10.4)	9.1 (8.0, 10.4)	9.3 (8.0, 10.6)	9.1 (8.0, 10.4)
2nd trimester	23.1 (20.0, 24.7)	22.6 (19.6, 24.6)	23.0 (19.9, 24.6)	23.0 (19.9, 24.6)	23.1 (20.0, 24.7)	22.9 (19.9, 24.6)
3rd trimester	30.9 (29.1, 34.6)	30.6 (29.1, 33.9)	30.9 (29.1, 34.7)	30.9 (29.1, 34.9)	30.9 (29.3, 34.6)	30.9 (29.1, 34.6)
**Gestational week at the last weight measurement, median (IQR)**						
1st trimester	9.6 (8.4, 11.0)	9.4 (8.1, 10.9)	9.3 (8.0, 10.6)	9.4 (8.1, 10.7)	9.4 (8.1, 10.7)	9.3 (8.1, 10.7)
2nd trimester	25.1 (24.0, 26.1)	24.9 (24.0, 26.0)	24.9 (24.0, 26.1)	24.9 (24.0, 26.0)	25.0 (24.1, 26.3)	24.9 (24.0, 26.0)
3rd trimester	37.1 (36.0, 38.4)	37.1 (36.0, 38.3)	37.1 (36.0, 38.3)	37.1 (36.0, 38.4)	37.0 (35.9, 38.3)	37.1 (36.0, 38.3)
**GWG (kg), mean (SD)**	10.8 (2.9)	7.8 (3.8)	14.8 (4.2)	11.6 (3.2)	8.9 (3.7)	15.0 (4.5)
**RGWG-T2 (kg/week), mean (SD)**	0.4 (0.1)	0.2 (0.2)	0.7 (0.2)	0.6 (0.2)	0.5 (0.3)	0.6 (0.3)
**RGWG-T2, %**						
Optimal	10,480 (100.0%)	NA	NA	3155 (23.3%)	2460 (24.2%)	4865 (14.3%)
Insufficient	NA	7647 (100.0%)	NA	2046 (15.1%)	2085 (20.5%)	3516 (10.3%)
Excessive	NA	NA	39,695 (100.0%)	8325 (61.5%)	5618 (55.3%)	25,752 (75.4%)
**RGWG-T3, kg/week, mean (SD)**	0.5 (0.2)	0.5 (0.3)	0.5 (0.2)	0.4 (0.1)	0.2 (0.2)	0.7 (0.2)
**RGWG-T3, %**						
Optimal	3155 (30.1%)	2046 (26.8%)	8325 (21.0%)	13,526 (100.0%)	NA	NA
Insufficient	2460 (23.5%)	2085 (27.3%)	5618 (14.2%)	NA	10,163 (100.0%)	NA
Excessive	4865 (46.4%)	3516 (46.0%)	25,752 (64.9%)	NA	NA	34,133 (100.0%)
**Child’s sex, %**						
Male	5256 (50.2%)	3748 (49.0%)	20,577 (51.8%)	6810 (50.3%)	5043 (49.6%)	17,728 (51.9%)
Female	5224 (49.8%)	3899 (51.0%)	19,118 (48.2%)	6716 (49.7%)	5120 (50.4%)	16,405 (48.1%)
**Maternal BMI at the first antenatal visit, %**						
Normal (18.5–25 kg/m^2^) (optimal range 0.35–0.50 kg/week)	8478 (80.9%)	4948 (64.7%)	25,549 (64.4%)	11,304 (83.6%)	7748 (76.2%)	19,923 (58.4%)
Underweight (> 18.5 kg/m^2^) (optimal range 0.44–0.58 kg/week)	490 (4.7%)	420 (5.5%)	855 (2.2%)	521 (3.9%)	735 (7.2%)	509 (1.5%)
Overweight (25–30 kg/m^2^) (optimal range 0.23–0.33 kg/week)	981 (9.4%)	1212 (15.8%)	10,071 (25.4%)	1209 (8.9%)	1106 (10.9%)	9949 (29.1%)
Obese (> 30 kg/m^2^) (optimal range 0.17– 0.27 kg/week)	531 (5.1%)	1067 (14.0%)	3220 (8.1%)	492 (3.6%)	574 (5.6%)	3752 (11.0%)
**Maternal age at birth (years), %**						
< 25	1043 (10.0%)	973 (12.7%)	3795 (9.6%)	1067 (7.9%)	821 (8.1%)	3923 (11.5%)
25–29	2657 (25.4%)	1972 (25.8%)	9957 (25.1%)	3105 (23.0%)	2128 (20.9%)	9353 (27.4%)
30–34	4010 (38.3%)	2724 (35.6%)	15,029 (37.9%)	5325 (39.4%)	3858 (38.0%)	12,580 (36.9%)
35–39	2311 (22.1%)	1605 (21.0%)	9098 (22.9%)	3391 (25.1%)	2708 (26.6%)	6915 (20.3%)
≥ 40	459 (4.4%)	373 (4.9%)	1816 (4.6%)	638 (4.7%)	648 (6.4%)	1362 (4.0%)
**Maternal smoking during pregnancy, %**						
No	9940 (94.8%)	7112 (93.0%)	37,168 (93.6%)	12,836 (94.9%)	9479 (93.3%)	31,905 (93.5%)
Yes	444 (4.2%)	491 (6.4%)	2132 (5.4%)	602 (4.5%)	608 (6.0%)	1857 (5.4%)
Missing	96 (0.9%)	44 (0.6%)	395 (1.0%)	88 (0.7%)	76 (0.7%)	371 (1.1%)
**Maternal birth region, %**						
Nordic	7985 (76.2%)	5390 (70.5%)	29,572 (74.5%)	10,317 (76.3%)	7451 (73.3%)	25,179 (73.8%)
Europe	557 (5.3%)	405 (5.3%)	2469 (6.2%)	739 (5.5%)	556 (5.5%)	2136 (6.3%)
Africa	459 (4.4%)	609 (8.0%)	1455 (3.7%)	544 (4.0%)	650 (6.4%)	1329 (3.9%)
Asia	1203 (11.5%)	1009 (13.2%)	5126 (12.9%)	1585 (11.7%)	1278 (12.6%)	4475 (13.1%)
Others	276 (2.6%)	233 (3.0%)	1069 (2.7%)	341 (2.5%)	228 (2.2%)	1009 (3.0%)
Missing	0 (0.0%)	NA (< 1%)	NA (< 1%)	0 (0.0%)	0 (0.0%)	NA (< 1%)
**Paternal birth region, %**						
Nordic	7928 (75.6%)	5297 (69.3%)	28,796 (72.5%)	10,165 (75.2%)	7330 (72.1%)	24,526 (71.9%)
Europe	597 (5.7%)	389 (5.1%)	2408 (6.1%)	793 (5.9%)	541 (5.3%)	2060 (6.0%)
Africa	475 (4.5%)	621 (8.1%)	1699 (4.3%)	580 (4.3%)	676 (6.7%)	1539 (4.5%)
Asia	1033 (9.9%)	952 (12.4%)	5066 (12.8%)	1475 (10.9%)	1167 (11.5%)	4409 (12.9%)
Others	325 (3.1%)	277 (3.6%)	1288 (3.2%)	372 (2.8%)	314 (3.1%)	1204 (3.5%)
Missing	122 (1.2%)	111 (1.5%)	438 (1.1%)	141 (1.0%)	135 (1.3%)	395 (1.2%)
**Maternal education level, %**						
Pre-highschool	954 (9.1%)	996 (13.0%)	3947 (9.9%)	1148 (8.5%)	1082 (10.6%)	3667 (10.7%)
High-school	3161 (30.2%)	2578 (33.7%)	13,413 (33.8%)	3996 (29.5%)	3127 (30.8%)	12,029 (35.2%)
Post-high school	6315 (60.3%)	4012 (52.5%)	22,163 (55.8%)	8317 (61.5%)	5892 (58.0%)	18,281 (53.6%)
Missing	50 (0.5%)	61 (0.8%)	172 (0.4%)	65 (0.5%)	62 (0.6%)	156 (0.5%)
**Household income quintiles at birth, %**						
First (lowest)	831 (7.9%)	814 (10.6%)	2920 (7.4%)	961 (7.1%)	887 (8.7%)	2717 (8.0%)
Second	1653 (15.8%)	1494 (19.5%)	6742 (17.0%)	2115 (15.6%)	1790 (17.6%)	5984 (17.5%)
Third	1663 (15.9%)	1294 (16.9%)	6676 (16.8%)	2065 (15.3%)	1673 (16.5%)	5895 (17.3%)
Fourth	2090 (19.9%)	1479 (19.3%)	8314 (20.9%)	2710 (20.0%)	1875 (18.4%)	7298 (21.4%)
Fifth (highest)	4235 (40.4%)	2552 (33.4%)	14,981 (37.7%)	5649 (41.8%)	3926 (38.6%)	12,193 (35.7%)
Missing	8 (0.1%)	14 (0.2%)	62 (0.2%)	26 (0.2%)	12 (0.1%)	46 (0.1%)
**Interpregnancy interval (years), %**						
First born	4857 (46.3%)	3404 (44.5%)	18,550 (46.7%)	5885 (43.5%)	3987 (39.2%)	16,939 (49.6%)
< 1	814 (7.8%)	678 (8.9%)	2654 (6.7%)	1033 (7.6%)	889 (8.7%)	2224 (6.5%)
1–2	1774 (16.9%)	1271 (16.6%)	6024 (15.2%)	2375 (17.6%)	1820 (17.9%)	4874 (14.3%)
2–5	1957 (18.7%)	1356 (17.7%)	7475 (18.8%)	2606 (19.3%)	2091 (20.6%)	6091 (17.8%)
5–10	454 (4.3%)	361 (4.7%)	2294 (5.8%)	713 (5.3%)	646 (6.4%)	1750 (5.1%)
> 10	116 (1.1%)	128 (1.7%)	640 (1.6%)	213 (1.6%)	182 (1.8%)	489 (1.4%)
Missing	508 (4.8%)	449 (5.9%)	2058 (5.2%)	701 (5.2%)	548 (5.4%)	1766 (5.2%)
**Maternal psychiatric history, %**	1049 (10.0%)	878 (11.5%)	4123 (10.4%)	1303 (9.6%)	1167 (11.5%)	3580 (10.5%)
**Hyperemesis gravidarum, %**	86 (0.8%)	140 (1.8%)	376 (1.0%)	122 (0.9%)	108 (1.1%)	372 (1.1%)
**Pre-eclampsia, %**	301 (2.9%)	293 (3.8%)	1488 (3.8%)	241 (1.8%)	188 (1.9%)	1653 (4.8%)
**Gestational diabetes mellitus, %**	22 (0.2%)	31 (0.4%)	127 (0.3%)	29 (0.2%)	48 (0.5%)	103 (0.3%)

### Statistical analysis

All statistical analyses were performed using Stata (version 16.0; StataCorp). For all models, we used cox regression models, clustered on maternal identification numbers and with robust standard errors to account for clustering of observations within mothers, to calculate hazard ratios (HRs) and 95% confidence intervals (CIs) for NDDs in offspring. We replaced the missing values in covariates as a dummy category for adjustment.

For continuous analyses, we fit models using restricted cubic splines models with 3 knots. The post-estimation command xbrcspline was used [22], with the reference value set as a *z*-score of 0 for the total GWG *z*-score analysis, 13.0 kg (median) for total GWG (kg), and 0.57 kg/week for RGWG-T2 and 0.51 kg/week for RGWG-T3, representing the median rates of GWG for each trimester. Analyses were repeated after stratification by maternal baseline BMI category. In model 1, HRs were adjusted for child’s sex and birth year. In model 2, we further adjusted for household income quintiles at birth, parental birth region, maternal age at birth, education level, IPI, baseline BMI, smoking during pregnancy, and psychiatric history. Each NDD outcome was modeled separately. *P*-values for analyses were calculated for a Wald test with a null hypothesis that all spline terms were jointly equal to 0, as a test of whether the exposure was generally associated with the outcome.

In categorical analyses, the “optimal” group was the reference group. Models were adjusted as above, with the exception of including maternal BMI in model 2, as the RGWG/GWG categories are conditioned on BMI. We assessed the proportionality assumption for Cox regression by including time/GWG category interaction terms in the fully adjusted models. When we found evidence showing hazard ratios changed over time with regard to NDDs in the cox regression models, we used flexible parametric survival models to plot the variance of HRs over time.

Multiple comparison adjustment with Bonferroni correction [23] was considered as the probability of identifying at least one significant result due to chance increases as more hypotheses are tested. The Bonferroni-adjusted significance level is 0.001 (based on 39 statistical comparisons in splines and categorical models).

We conducted several sensitivity analyses. Analyses of total GWG *z*-score and any NDD diagnosis were repeatedly stratified by offspring sex (given the theory that the high male:female ratios among those diagnosed with NDDs may relate to differing etiological pathways) and restricted to Nordic-born mothers (as ethnic groups represented among those who are immigrants to Sweden may differ in GWG patterns [24] and also have different patterns of NDD diagnoses). Since our observations indicated that the risk of NDDs was associated with elevated third trimester weight gain, we repeated our analyses of maternal RGWG-T3 after excluding women diagnosed with pre-eclampsia or gestational diabetes mellitus (GDM), as pre-eclampsia and GDM may induce rapid weight gain in later pregnancy and were also associated with offspring risk of NDDs [25, 26]. As hyperemesis gravidarum may induce slow weight gain in early pregnancy and was also associated with NDDs [27, 28], we repeated our analyses of maternal RGWG-T2 after excluding women diagnosed with hyperemesis gravidarum. As we observed 5.2% of the children had missing values in maternal IPI, we repeated our analyses after excluding those with missing values in IPI. Furthermore, the number of antenatal visits may be influenced by factors such as pregnancy complications which could then influence the accuracy of the RGWG calculation. An accelerated fetal growth usually occurs in the late second trimester [29], which is also a component of maternal gestational weight gain. Therefore, we repeated our analysis between RGWG-T2 and NDDs by additionally adjusting for the number of antenatal visits in the second trimester and performed the stratification analyses among those with the last weight measured < 25 and ≥ 25 weeks of gestation in the second trimester. As we found that the excluded and included populations differed in several characteristics, we repeated our analyses after applying inverse probability weights (IPW) to correct the analysis by weighting the observations with the probability of being selected [30].

## Results

### Study sample

**Fig. 1:**
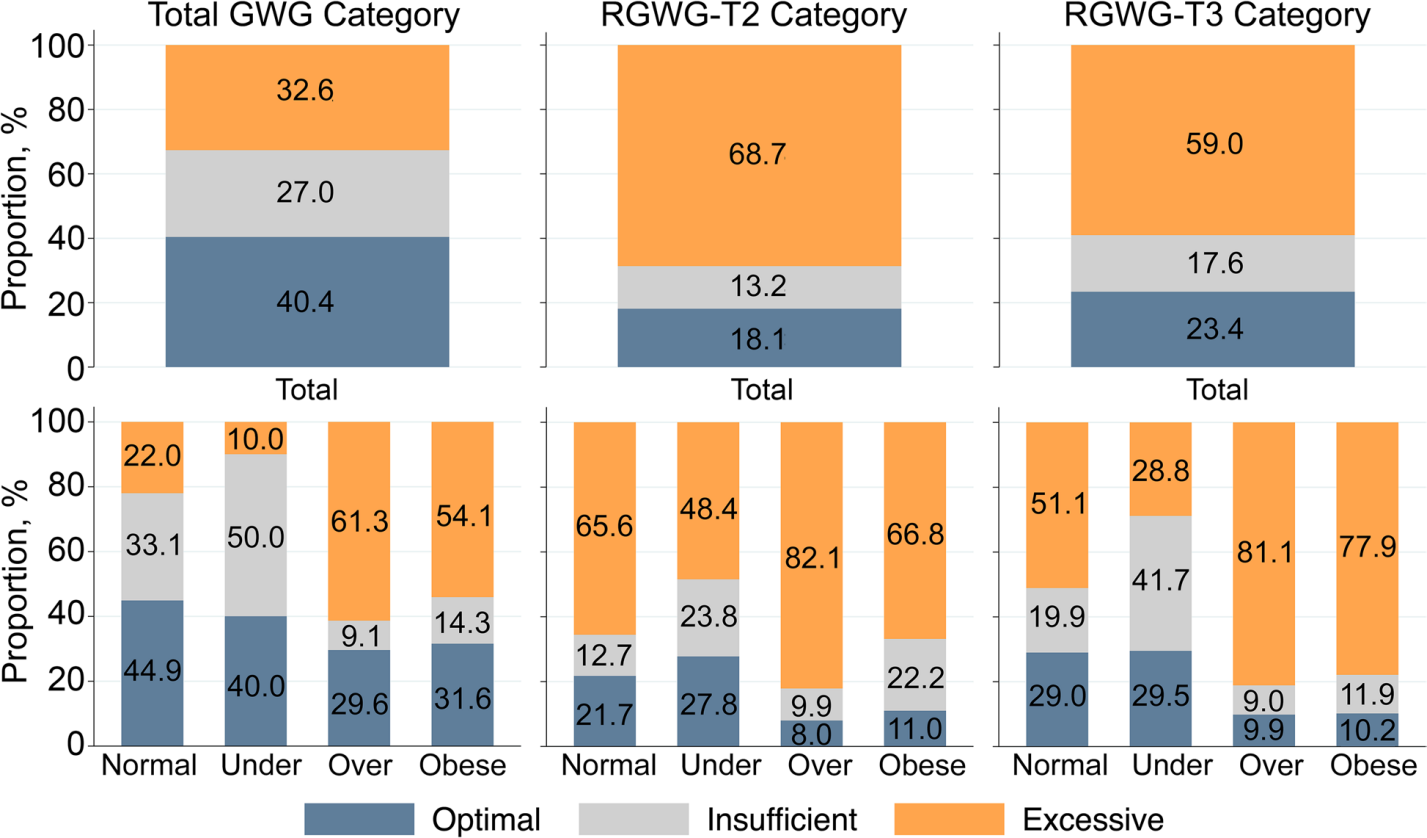
Distributions of total GWG (kg), RGWG-T2, and RGWG-T3 categories according to the IOM guidelines

Of the total sample of 57,822 children (29,581 [51.2%] male; mean [SD] follow-up time after 2 years of age, 5.4 [1.1] years), 2205 (3.8%) received an NDD diagnosis by the end of the follow-up. The majority of children (67.4%) were born to mothers with baseline BMI within the normal range, whereas 29.5% of mothers were overweight or obese. Most mothers gained a total amount of weight outside of the optimal range: 33% and 27% of women gained excessive and inadequate total amounts of weight during pregnancy, respectively (Fig. 1).

Compared with optimal RGWG groups, mothers who exceeded the GWG guidelines were more likely to be primiparous, carrying a male fetus, younger than 30 years, or born outside of Nordic countries; to have lower family income, lower education level, and a history of psychiatric history; and to report smoking in early pregnancy (Table 1). We observed a similar pattern for total GWG categories (Additional file 1: Table S3).

### Total GWG and risk of NDDs

**Fig. 2:**
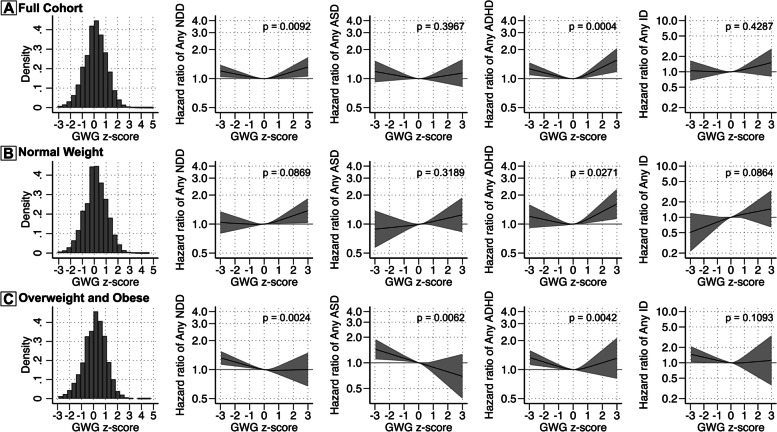
Maternal *z*-score for gestational weight gain (GWG) and offspring risk for neurodevelopment disorders in the full cohort (**A**) and according to the category of maternal BMI at first antenatal visit (**B**, **C**). Histograms illustrate the distribution of GWG *z*-score for those included in each analysis. Adjusted estimates are shown for any NDD, ASD, ADHD, and ID. The curved solid black line represents the hazard ratio (HR) calculated through restricted cubic splines models with 3 knots. The grey bands represent the 95% CI. A reference line is included for an HR of 1.00. *P*-values for analyses are shown for a Wald test with a null hypothesis that all spline terms were jointly equal to 0, as a test of whether the exposure was generally associated with the outcome. The model was adjusted for birth year, child’s sex, maternal age at birth, household income quintiles at birth, maternal education level, parental birth region, interpregnancy interval, maternal psychiatric history, maternal smoking during pregnancy, and maternal BMI at first antenatal visit (only in the full cohort analysis). Note that the *y*-scale differs for ID compared to the other outcomes

Examining GWG *z*-scores (accounting for the length of gestation), we observed J-shaped associations of GWG *z*-scores with any NDDs and ADHD, with slightly stronger associations for higher GWG compared to a lower GWG (Fig. 2A). For example, a total GWG of two standard deviations above the referent of 0 (GWG *z*-score = 2) was associated with 19% increased risk of any NDD diagnosis (95% CI = 1.03–1.37) and 31% increased risk of any ADHD diagnosis (95% CI = 1.10–1.57), which were higher compared to the associations with a total GWG of two standard deviations below the referent of 0 (GWG *z*-score = − 2) (12% for any NDDs, 95% CI = 1.02–1.23; 15% for ADHD, 95% CI = 1.05–1.27). To put this into context, a GWG *z*-score of 2 and − 2 in our cohort would correspond to a total weight gain of 25.9 and 6.8 kg for normal-weight women delivering at 40 weeks, respectively, compared to 14.2 kg corresponding to *z* = 0 for the same group. However, only the association with ADHD survived Bonferroni correction. When stratified by maternal baseline BMI, the associations between higher GWG *z*-scores and the risks for NDDs and ADHD remained (Fig. 2B), but results showed wide CIs for the associations with lower GWG *z*-scores in the normal BMI group (Fig. 2C). Among overweight and obese women, lower maternal GWG *z*-scores were associated with increased risks of any NDDs, ASD, and ADHD, but results showed wide CIs for the associations with higher GWG *z*-scores (Fig. 2C).

We observed steeper U-shaped associations of maternal GWG with offspring risk of any NDDs and any ADHD when we used the original values of total GWG (in kilograms; without adjustment for length of gestation) (Additional file 1: Fig. S3), while analysis of categories based on IOM recommendations for total weight gain did not indicate any associations with offspring risk of NDDs after adjustment for confounders (Additional file 1: Table S4).

### Rates of GWG in the second trimester and risk of NDDs

**Fig. 3:**
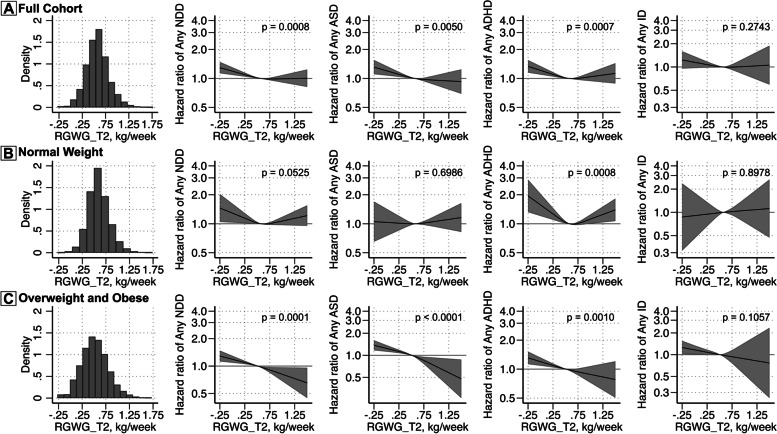
Rate of gestational weight gain during the second trimester (RGWG-T2) and offspring risk for neurodevelopment disorders in the full cohort (**A**) and according to the category of maternal BMI at first antenatal visit (**B**, **C**). Histograms illustrate the distribution of RGWG-T2 for those included in each analysis. Adjusted estimates are shown for any NDD, ASD, ADHD, and ID. The curved solid black line represents the hazard ratio (HR) calculated through restricted cubic splines models with 3 knots. The grey bands represent the 95% CI. A reference line is included for an HR of 1.00. *P*-values for analyses are shown for a Wald test with a null hypothesis that all spline terms were jointly equal to 0, as a test of whether the exposure was generally associated with the outcome. The model was adjusted for birth year, child’s sex, maternal age at birth, household income quintiles at birth, maternal education level, parental birth region, interpregnancy interval, maternal psychiatric history, maternal smoking during pregnancy, and maternal BMI at first antenatal visit (only in the full cohort analysis)

In the continuous analyses, lower RGWG-T2 was associated with increased risk for any NDDs, ASD, and ADHD (Fig. 3A). For example, maternal weight gain of 0.25 kg/week was associated with a 9% increased risk of any NDD diagnosis (95% CI = 1.04–1.15) compared to the median of 0.57 kg/week in the fully adjusted model. Only the associations with any NDDs and ADHD survived the Bonferroni correction. When stratified by baseline maternal BMI category, the associations remained largely similar, although with wider CIs (Fig. 3B, C) and with higher point estimates associated with lower RGWG-T2 among normal-weight mothers for risk of any ADHD. However, increasing RGWG-T2 above the median was associated with an increased risk of ADHD among children to normal-weight mothers and a marginally lower risk of ASD among children to overweight/obese mothers.

In the 3-category RGWG-T2 analysis, compared to those with an optimal rate of weight gain during the second trimester, insufficient maternal RGWG-T2 was associated with increased risk of any ADHD diagnosis (1.30, 1.08–1.57) and specifically ASD with ADHD (1.75, 1.19–2.57) in fully adjusted models (Additional file 1: Table S5). However, RGWG-T2 was not associated with other NDDs or mutually exclusive diagnoses. In the 5-category RGWG-T2 analysis, extremely insufficient and insufficient RGWG-T3 were associated with 35% (1.35, 1.07–1.70) and 26% (1.26, 1.01–1.57), respectively, increased risk of any ADHD while none of them survived the Bonferroni correction. However, we did not observe any associations of excessive or extremely excessive RGWG-T2 with any NDD diagnoses (Table 2). We did not observe any indication of interaction between RGWG-T2 and follow-up time, with exception of models for ADHD, which indicated potential increases in risk associated with maternal excessive RGWG-T2 as children grew older (Additional file 1: Fig. S4).

**Table 2:** Associations between the rate of gestational weight gain at different stages of pregnancy and offspring risks of neurodevelopment disorders in the full cohort

Extended category^**a**^	RGWG–T2					RGWG–T3				
	***N*** cases	%^**b**^	Model 1^**c**^	Model 2^**d**^	***P***-value	***N*** cases	%^**b**^	Model 1^**c**^	Model 2^**d**^	***P***-value^e^
**Optimal**										
Any NDDs	380	3.63	1.00 (ref)	1.00 (ref)	–	469	3.47	1.00 (ref)	1.00 (ref)	–
Any ASD	207	1.98	1.00 (ref)	1.00 (ref)	–	253	1.87	1.00 (ref)	1.00 (ref)	–
Any ADHD^f^	217	2.07	1.00 (ref)	1.00 (ref)	–	278	2.06	1.00 (ref)	1.00 (ref)	–
Any ID	42	0.40	1.00 (ref)	1.00 (ref)	–	51	0.38	1.00 (ref)	1.00 (ref)	–
**Extremely insufficient**										
Any NDDs	160	4.80	**1.35 (1.12–1.62)**	1.16 (0.96–1.40)	0.12	165	3.61	1.05 (0.88–1.26)	0.99 (0.83–1.18)	0.91
Any ASD	76	2.28	1.18 (0.90–1.53)	1.07 (0.82–1.39)	0.64	83	1.82	0.99 (0.77–1.27)	0.95 (0.74–1.21)	0.66
Any ADHD^f^	110	3.30	**1.62 (1.29–2.04)**	**1.35 (1.07–1.70)**	0.01	107	2.34	1.16 (0.92–1.45)	1.08 (0.86–1.35)	0.52
Any ID	23	0.69	**1.76 (1.06–2.92)**	1.42 (0.85– 2.39)	0.18	15	0.33	0.88 (0.49–1.56)	0.76 (0.43–1.35)	0.35
**Insufficient**										
Any NDDs	183	4.24	1.19 (1.00–1.42)	1.12 (0.94–1.34)	0.20	178	3.18	0.91 (0.77–1.09)	0.89 (0.75–1.06)	0.18
Any ASD	88	2.04	1.06 (0.82–1.36)	1.02 (0.79–1.31)	0.89	91	1.63	0.87 (0.68–1.10)	0.85 (0.67–1.08)	0.17
Any ADHD^f^	120	2.78	**1.36 (1.09–1.70)**	**1.26 (1.01–1.57)**	0.04	111	1.98	0.96 (0.77–1.19)	0.93 (0.75–1.16)	0.53
Any ID	19	0.44	1.12 (0.65–1.92)	1.02 (0.59–1.75)	0.96	25	0.45	1.18 (0.73–1.90)	1.08 (0.67–1.73)	0.77
**Excessive**										
Any NDDs	719	3.65	0.99 (0.87–1.12)	0.98 (0.86–1.11)	0.70	677	3.57	1.02 (0.91–1.15)	0.98 (0.87–1.10)	0.69
Any ASD	373	1.89	0.94 (0.79–1.12)	0.93 (0.78–1.10)	0.40	350	1.84	0.98 (0.84–1.16)	0.95 (0.81–1.11)	0.51
Any ADHD^f^	420	2.13	1.01 (0.86–1.19)	0.99 (0.85–1.17)	0.95	398	2.10	1.01 (0.87–1.18)	0.96 (0.82–1.12)	0.62
Any ID	97	0.49	1.22 (0.85–1.75)	1.21 (0.84–1.74)	0.31	90	0.47	1.26 (0.89–1.77)	1.19 (0.84–1.67)	0.33
**Extremely excessive**										
Any NDDs	763	3.81	1.03 (0.91–1.16)	0.97 (0.86–1.10)	0.65	716	4.72	**1.34 (1.19–1.51)**	**1.17 (1.04–1.32)**	0.01
Any ASD	375	1.87	0.93 (0.78–1.10)	0.89 (0.75–1.05)	0.16	342	2.26	**1.19 (1.01–1.40)**	1.08 (0.91–1.27)	0.39
Any ADHD^f^	486	2.43	1.14 (0.97–1.34)	1.07 (0.91–1.25)	0.43	459	3.03	**1.46 (1.26–1.69)**	**1.23 (1.06–1.43)**	0.01
Any ID	89	0.44	1.10 (0.76–1.58)	1.06 (0.73–1.52)	0.78	89	0.59	**1.56 (1.10–2.20)**	**1.44 (1.01–2.03)**	0.04

*Abbreviations*: *RGWG* rate of gestational weight gain, *Ref* reference, *NDDs* neurodevelopmental disorders, *ASD* autism spectrum disorder, *ADHD* attention deficit/hyperactivity disorder, *ID* intellectual disability

^a^For normal weight women, the optimal rate of weight gain during the second and third trimesters was 0.35–0.50 kg/week, the extremely insufficient rate was < 0.27 kg/week, the insufficient rate was 0.27–< 0.35 kg/week, the excessive rate was > 0.50–0.68 kg/week, and the insufficient rate was > 0.68 kg/week. For underweight women, the optimal rate was 0.44–0.58 kg/week, the extremely insufficient rate was < 0.37 kg/week, the insufficient rate was 0.37–< 0.44kg/week, the excessive rate was > 0.58–0.72 kg/week, and the insufficient rate was > 0.72 kg/week. For overweight women, the optimal rate was 0.23–0.33 kg/week, the extremely insufficient rate was < 0.12 kg/week, the insufficient rate was 0.12–< 0.23 kg/week, the excessive rate was > 0.33–0.61 kg/week, and the insufficient rate was > 0.61 kg/week. For obese women, the optimal rate was 0.17–0.27kg/week, the extremely insufficient rate was < 0 (weight loss) kg/week, the insufficient rate was 0–< 0.17 kg/week, the excessive rate was > 0.27–0.51 kg/week, and the insufficient rate was > 0.51 kg/week

^b^Calculated as the number of cases observed when following children from 2 years of age for a mean [SD] of 5.4 [1.1] years, divided by the number of children at risk for developing the disorder

^c^Model 1: Cox regression model, clustered on the maternal identifier, adjusted only for birth year and child’s sex. Results are displayed as the hazard ratio (95% confidence interval)

^d^Model 2: Cox regression model, clustered on the maternal identifier, adjusted for birth year, child’s sex, maternal age at birth, household income quintiles at birth, maternal education level, parental birth region, interpregnancy interval, maternal psychiatric history, and maternal smoking during pregnancy. Results are displayed as the hazard ratio (95% confidence interval)

^e^*P*-values for model 2

^f^An interaction with time was observed for these categories, indicating that the HR changes over time (see Additional file 1: Fig. S4)

### Rates of GWG in the third trimester and risk of NDDs

**Fig. 4:**
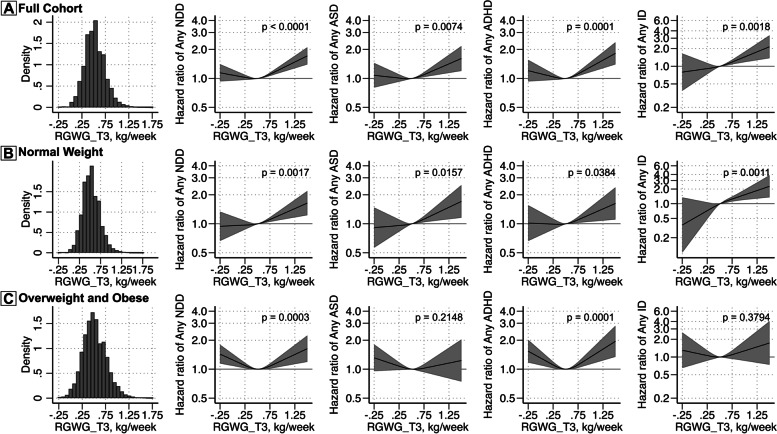
Rate of gestational weight gain during the third trimester (RGWG-T3) and offspring risk for neurodevelopment disorders in the full cohort (**A**) and according to category of maternal BMI at first antenatal visit (**B**, **C**). Histograms illustrate the distribution of RGWG-T3 for those included in each analysis. Adjusted estimates are shown for any NDD, ASD, ADHD, and ID. The curved solid black line represents the hazard ratio (HR) calculated through restricted cubic splines models with 3 knots. The grey bands represent the 95% CI. A reference line is included for an HR of 1.00. *P-*values for analyses are shown for a Wald test with a null hypothesis that all spline terms were jointly equal to 0, as a test of whether the exposure was generally associated with the outcome. The model was adjusted for birth year, child’s sex, maternal age at birth, household income quintiles at birth, maternal education level, parental birth region, interpregnancy interval, maternal psychiatric history, maternal smoking during pregnancy, and maternal BMI at first antenatal visit (only in the full cohort analysis). Note that the *y*-scale differs for ID compared to the other outcomes

In the continuous analysis, in contrast to findings for RGWG-T2, no association was apparent between lower maternal RGWG-T3 and offspring risk of NDD outcomes (Fig. 4), nor was there any indication that insufficient maternal RGWG-T3 was associated with offspring risk of NDDs in the categorical analysis (Table 2). A pattern of increasing risk with higher RGWG-T3 was observed for all outcomes (Fig. 4A), with a rate of 1 kg/week associated with a 28% increased risk of any diagnosis (95% CI = 1.16–1.40), 24% increased risk of ASD (95% CI = 1.08–1.43), 31% increased risk of ADHD (95% CI = 1.16–1.48), and 44% increased risk of ID diagnoses (95% CI = 1.17–1.77), compared to the median of 0.51 kg/week. However, only the associations for any NDD and for ADHD survive Bonferroni correction. Similar patterns were observed for women after stratification on baseline maternal BMI, though with wider confidence intervals for estimates among overweight/obese mothers. However, decreasing RGWG-T3 below the median was also associated with an increased risk of any NDDs and ADHD among women who were overweight or obese (Fig. 4B, C). In categorical analyses, compared to those with an optimal weight gain, extremely excessive RGWG-T3 was associated with an increased risk of any NDD diagnosis, any ADHD, and any ID (Table 2). We did not find any associations between excessive RGWG-T3 and any NDD diagnoses or mutually exclusive diagnoses (Additional file 1: Table S5). We did not observe any indication of interaction between RGWG-T3 and follow-up time, with exception of models for ADHD, which indicated potential increases in risk associated with maternal extremely excessive RGWG-T3 as children grew older (Additional file 1: Fig. S4).

### Rates of GWG in the second and third trimesters and risk of NDDs

Compared to those with optimal rate of GWG in both second and third trimesters (Additional file 1: Table S6), insufficient maternal RGWG in the second trimester but excessive RGWG in the third trimester was associated with increased risk of ADHD (1.55, 1.13–2.13) and ID (2.53, 1.15–5.55).

### Sensitivity analyses

After stratification by sex, higher GWG *z*-scores were associated with increased risk for any NDDs and ADHD in male offspring, though the patterns for the point estimates were generally similar among females. Lower GWG *z*-scores were associated with any NDDs, ASD, and ADHD in female offspring (Additional file 1: Fig. S5). However, there was no evidence for interaction by sex (all *P*-values for interaction > 0.05). Similar patterns of associations were observed compared to the primary analyses when analyses were restricted to Nordic-born mothers (Additional file 1: Fig. S6). The association of higher RGWG-T3 with increased offspring risk of any NDDs and any ADHD remain unchanged when restricted to mothers without pre-eclampsia or GDM, and the associations of lower RGWG-T2 with any NDDs, any ASD, and any ID remained unchanged when restricted to mothers without hyperemesis gravidarum (Additional file 1: Fig. S7). Furthermore, excluding those with missing values in IPI did not change the main results (Additional file 1: Fig. S8). Moreover, we found the associations were similar to the main results when adjusting for the number of antenatal visits in the second trimester or restricting the population to those with the last weight measured < 25 weeks in the second trimester (Additional file 1: Fig. S9B&C). The relationship between lower RGWG-T2 and higher risk for any NDDs, ASD, and ADHD remained when restricting the population to those with last weight measured ≥ 25 weeks in the second trimester, while we found a higher RGWG-T2 was associated with higher risks for ADHD, even though after the adjustment for GDM and pre-eclampsia (Additional file 1: Fig. S9D&E). Finally, after applying the inverse probability weights (IPW) to correct the analysis by weighting the observations with the probability of being selected, we found the impact of selection bias was negligible (Additional file 1: Fig. S10).

## Discussion

In this population-based cohort study, we observed J-shaped associations between total GWG and offspring risks of any NDDs, particularly ADHD, using a *z*-score measure that accounted for length of gestation. The associations between rates of weight gain and NDDs in offspring varied by the timing of weight gain during pregnancy and differed with regard to specific NDD diagnoses. Lower RGWG during the second trimester was associated with an increased risk of any NDDs in offspring, particularly ASD and ADHD, while higher RGWG during the third trimester was associated with a higher risk of all three NDD diagnoses examined. When rates of weight gain in the second and third trimesters were considered together, we found that insufficient weight gain in the second trimester followed by excessive weight gain in the third trimester was most significantly associated with increased risks of ADHD and ID in offspring.

### Comparison with previous studies

The proportions of total gestational weight gain and rate of gestational weight gain in the second and third trimesters in our study were comparable to the findings in previous studies that also relied on the IOM guidelines [31, 32]. To our knowledge, two previous studies have investigated the relationship between the rate of GWG and the risk of NDD outcomes. In a cohort study including 12,556 children, Rodriguez et al. reported that rates of weekly weight gain (calculated using observations over the entire pregnancy) were not significantly associated with teacher-reported ADHD symptoms in offspring among normal weight or underweight women but were associated with increased offspring odds of ADHD symptoms among women with high-pregnancy BMI [33]. In a case-control study including 4409 children, Matias et al. calculated the rates of GWG for the second and third trimesters together and found that RGWG below or above the optimal range according to the IOM guidelines did not significantly increase the risks of ASD or developmental delay after adjusting for confounders, though point estimates for ORs for ASD and developmental delay were above one for excessive GWG categories [34]. A key difference between these studies and our current study is in the treatment of the rates of GWG. We observed different patterns when considering RGWG in the second and third trimesters separately, and we also took the non-linear associations with NDDs into consideration. However, previous studies considered only an overall rate of weight gain and assessed a linear relationship with NDDs. Such variations suggested different effects of weight gain on fetal neurodevelopment during specific timing of exposure.

Existing studies relating to total GWG and NDDs have used different definitions for GWG as well as outcomes, which in turn influences the comparability of their results. In previous studies, autism was the most commonly considered outcome, and IOM guidelines were most frequently used to identify non-optimal GWG, followed by treating total GWG as a continuous variable. In a recent meta-analysis, evidence from five cohort studies and four case-control studies (involving 323,253 participants) showed that both excessive and inadequate GWG (according to IOM guidelines 2009 [8 studies]/1990 [1 study]) were associated with a higher risk of ASD in offspring [7]. Matias et al. reported that the GWG *z*-score in the highest tertile was associated with 22% higher odds of ASD after adjustment for confounders while no significant associations were found with regard to the lowest tertile of GWG *z*-scores [34]. We also observed a U-shaped pattern of association between total GWG (kg) and children’s risk of NDDs, but with wide confidence intervals for the outcome of ASD. The U-shape was attenuated when the length of gestation was taken into account (i.e., GWG *z*-score), especially at the left tail which represented insufficient GWG.

Few studies have focused on ADHD and ID in relation to total weight gain, and their results are often inconsistent. In a cohort study involving 331 children, Fuemmeler et al. reported that GWG below IOM recommendations was associated with hyperactive-impulsive symptoms in offspring and GWG above recommendation was associated with worsened working memory, planning and organizing behavior in offspring between 2 and 6 years old [9]. However, two other cohort studies (involving 12,556 children and 511 children respectively) found no significant associations between GWG (categorized according to the IOM guidelines 1990 or GWG *z*-scores) and ADHD symptoms [33, 35]. In our study, we observed an apparent U-shaped association between total GWG (kg) and ADHD, while the association between lower GWG and the risk of ADHD in offspring was largely attenuated when the length of gestation (i.e., GWG *z*-score) was accounted for.

Among 78,675 children, Mann et al. reported gestational weight change (gain or loss) was not significantly associated with the odds of ID [36]. However, in a Swedish register-based study involving 467,485 children, Lee et al. indicated that inadequate GWG (according to the IOM guidelines) may increase the risk of ID in offspring, regardless of maternal BMI and such associations remained after excluding children born preterm [8]. We did not observe an apparent association between total GWG (kg) and ID in offspring, though this may be due to our limited sample size. A novel finding in our study is that children’s risk of ID was most pronounced for women who experienced insufficient weight gain in the second trimester followed by excessive weight gain in the third trimester. While this finding requires confirmation with larger study samples, it suggests that studies of GWG in relation to children’s risk for ID may need to consider the rate of weight gain over time in addition to the total amount gained.

In this study, the associations between total GWG and NDDs from continuous analyses were more pronounced than categorical analyses based on the IOM guidelines. The results in the categorical analyses should be interpreted with caution as none of them survived Bonferroni correction. However, it should be noted that the Bonferroni adjustment may be overly conservative [37], as this approach decreases the risk of false positive results (type I errors) at the cost of increasing the risk of false negative results (type II errors). Our findings suggest that studying the full range of continuous GWG values might better capture the risk associated with NDDs, for both total GWG and rates of weight gain, in line with recent good practice recommendations for studies of GWG in observational studies [10]. The associations we observed between excessive total GWG with NDDs were generally consistent when comparing total GWG in kg to GWG *z*-scores accounting for pregnancy durations. However, the associations of insufficient GWG with NDDs were largely attenuated when considering GWG *z*-scores. This finding was in line with other studies investigating perinatal outcomes [38]. Since total GWG and NDD outcomes are highly correlated with gestational duration, the use of GWG *z*-score enabled us to disentangle the associations with pregnancy weight gain from the effects of the gestational duration.

### Potential mechanisms

The association between excessive GWG and fetal neurodevelopment may be related to the downstream effect of increased maternal/fetal adipose tissues. A number of plausible pathways to link increased maternal or fetal adiposity to alternations in neurodevelopment have been hypothesized, including dysregulated pro-inflammatory cytokine signaling; lipotoxicity; increased oxidative stress, dysregulated insulin, glucose, and leptin signaling; dysregulated serotonergic and dopaminergic signaling; and perturbations in synaptic plasticity [39, 40]. Furthermore, excessive or rapid GWG may also be related to gestational diabetes or pathological edema caused by preeclampsia, which has also been associated with increased risks of NDDs in offspring [25, 26]. Finally, excessive GWG is also associated with macrosomia and LGA fetuses, which are associated with greater risks of asphyxia-related complications during labor [41] and increased risk of NDDs [42, 43].

There are two potential hypotheses for linking insufficient GWG to NDDs in offspring [7]: (1) insufficient GWG may be considered a marker of maternal nutritional deficiency which in turn causes suboptimal nutritional states in the developing fetus, detrimentally influencing fetal brain development [44], and (2) insufficient GWG can be associated with co-morbidities during pregnancy such as anorexia nervosa, hyperemesis gravidarum, and intestinal malabsorption which could lead to maternal nutrient deficiencies and placental dysfunction-related complications [45, 46]. Insufficient GWG is also associated with higher risks for low birth weight and preterm birth [31] which are themselves associated with higher risks of NDDs.

We observed that insufficient RGWG during the second trimester and excessive RGWG during the third trimester were associated with increased risk of NDDs, in line with the notion that the effect of any obstetric-related factors (with regard to nutrient deficiency or overload) on fetal neurodevelopment depends on the timing of exposure [42, 44]. Considering RGWG-T2 and RGWG-T3 together, we found that insufficient RGWG during the second trimester and excessive RGWG during the third trimester were most significantly associated with increased risks of NDDs (especially for ADHD and ID), which could be related to a double jeopardy effect stemming from these perturbations. One potential condition related to this phenomenon could be hyperemesis gravidarum. Mothers with hyperemesis gravidarum (severe nausea and vomiting) usually have slower weight gain or lose weight in early pregnancy while a “catch-up” weight gain may occur later in pregnancy as this condition usually resolves after 20 weeks of gestation [27, 47]. Hyperemesis gravidarum has been associated with increased risks of ASD, ADHD, and cognitive impairment of offspring [28]. While hyperemesis gravidarum could plausibly be related to the associations that we observe, our sensitivity analyses indicate that the associations between low gestational weight gain in the second trimester and children’s risk of NDDs cannot be entirely explained by this condition.

In this study, we observed sex differences in the associations which suggest that fetal vulnerability to aberrant maternal metabolic and nutritional states may differ by fetal sex, with higher risk for any NDD and particularly autism among females associated with lower total maternal weight gain, though no interactions were detected in formal testing. Female fetuses have a higher survival rate than male fetuses during periods of maternal malnutrition, which has been observed under very harsh conditions, such as during the Dutch famine period [48]. We also noted that the proportion of female children is slightly higher among mothers with insufficient weight gain compared to other categories. In the Dutch famine cohort, sex differences in certain neurodevelopmental outcomes have been reported, with exposure to early prenatal famine associated with a higher incidence of spina bifida only in males, but more strongly associated with other neurodevelopmental conditions, such as epilepsy, cerebral palsy, and spastic diplegia, among females [49]. Our observations in the sex stratification analysis are in line with the notion that female fetuses generally have higher survival rates than male fetuses under stress conditions, though remain vulnerable to the influences of maternal undernutrition on neurodevelopment.

### Strengths and limitations

An important strength of our study is that we not only used the IOM guidelines, but also used gestational age-standardized GWG *z*-scores to define total GWG which disentangled the effect of gestational duration from that of GWG. This measure was developed specifically for the Swedish population using similar register resources as were available in this study [11] and provides *z*-score measures for all BMI categories, though other international methods to estimate GWG *z*-scores indicate similar weight gain patterns (e.g., the INTERGROWTH-21 charts indicate a weight gain of 24.6 kg for normal weight women at 40 weeks if *z* = 2 compared to 13.7 kg if *z* = 0) [50]. Using maternal weight data taken from multiple time periods during pregnancy, we were able to explore the critical windows of development during which non-optimal weight change may have the greatest detrimental effect on fetal neurodevelopment. For weight gain during the second and third trimesters, we calculated RGWG as weight gain divided by the number of interval weeks to reduce bias due to the length of observation [10, 51]. We used objectively measured, prospectively recorded data from Swedish registry data to define exposures, outcomes, and covariates to minimize the possibility of bias. Finally, important potential confounders, such as maternal BMI and maternal psychiatric history, were accounted for in the analyses.

Some limitations in this study should also be mentioned. First, maternal weight measured during the first antenatal visit is a pragmatic but insufficient proxy measure for pre-pregnancy BMI. This method may have overestimated pre-pregnancy BMI because of weight gain that occurred between conception and the first antenatal visit (median gestational age of 9 weeks in this study). However, weight gain within the first trimester is minimal in most cases [52]. Second, random errors in the measurement of weight may exist in our study because we used weight data collected across multiple clinics. These errors may have diminished the strength of the observed HRs. Third, we were unable to separately explore the association between GWG and the risk of NDDs among underweight and obese mothers because of limited sample sizes. However, metabolic or nutritional disturbances may be of greater importance in these populations. Fourth, limitations in sample sizes and follow-up time in this study could be a potential issue for investigating the relationships between maternal weight gain and offspring risks of NDDs due to the low prevalence of NDDs, especially for ASD and ID. Limited follow-up time compared to other register-based studies likely resulted in the misclassification of children who will eventually receive NDD diagnoses, biasing our estimates toward the null. Future studies with larger sample sizes and longer follow-up times are warranted to replicate our findings. Fifth, the baseline characteristics differed in the included and excluded population in our study which may indicate selection biases, though the impact of such selection bias appears to be negligible. Additionally, although we have accounted for many confounders, residual confounding may still exist, such as specific components of the maternal diet or genetic predisposition. We were unable to carry out sibling comparisons or other family-based study designs to address this issue, given the limited sample size and number of birth years for which GWG data were available. Furthermore, we did not have biomarkers of intermediate conditions (e.g., inflammation, endocrine alterations) that may help elucidate the underlying mechanisms connecting maternal GWG with offspring risk of NDDs. Finally, our study population was dominated by Nordic-born mothers. Therefore, our findings would need to be replicated in other populations to verify their generalizability.

## Conclusions

During pregnancy, most women gain weight outside of the optimal range commonly recommended by clinicians. Here, we report that insufficient rates of weight gain during the second trimester and excessive rates of weight gain during the third trimester were associated with a higher risk of NDD outcomes, suggesting that intensity (the rate of GWG) and timing of exposure (at different stages of pregnancy) also play an important role. In addition, by accounting for gestational durations, we showed J-shaped associations between total GWG and risks of NDDs in offspring, especially for ADHD. These results require replication in larger and more diverse populations. Future studies with more specific assessments of genetic and metabolic factors responsible for insufficient and excessive GWG during pregnancy are also warranted.

## Supplementary Information

**Additional file 1: Table S1.** Included and excluded individuals**.** The characteristics of the included and excluded population in our study. **Table S2.** Diagnostic codes. The codes for identifying exposures, outcomes, and covariates. **Table S3.** Study cohort description by RGWG category. The characteristics of the study sample according to different categories of RGWG. **Table S4.** Total GWG category and offspring risk of NDCs in full cohort. The association between the 3-category total GWG and offspring risks of autism, ADHD and ID, and the mutuality exclusive diagnoses of these three NDCs. **Table S5.** RGWG category and offspring NDCs in full cohort. The association between the 3-catgory total RGWG and offspring risks of autism, ADHD and ID, and the mutuality exclusive diagnoses of these three NDCs. **Table S6.** RGWG in second and third trimester together with offspring NDCs in full cohort. The association between RGWG in second and third trimester in combination and offspring risks of NDCs. **Fig. S1.** Sample derivation and outcome description. A description of sample derivation and NDC co-occurrence in offspring. **Fig. S2.** Directed acyclic graph. A directed acyclic graph describing how potential confounders influence the exposures and outcomes. **Fig. S3.** Total GWG (kg) and offspring NDCs. Spline models depicting the association between GWG (kg) and offspring NDCs. **Fig. S4.** Visualization for the change of hazard ratios over time. We observed evidence in the cox regression models that the hazard ratios may be time varying for the association between excessive RGWG-T2 and any ADHD, and the association between extremely excessive RGWG-T3 and any ADHD. We therefore visualized the hazard ratios over time by using flexible parametric survival models for non-linear time-dependent effects. **Fig. S5.** Total GWG z-scores and offspring NDCs (sex stratification). Spline models depicting the association between total GWG z-scores and offspring NDCs, stratified by child’s sex. **Fig. S6.** Total GWG z-scores and offspring NDCs (restricted to Nordic-born mothers). Spline models depicting the association between total GWG z-scores and offspring NDCs, restricted to Nordic-born mothers. **Fig. S7.** RGWG and offspring NDCs (restricted to non-hyperemesis gravidarum, non-preeclampsia or non-gestational diabetes mellitus). Spline models depicting the association between total RGWG and offspring NDCs, restricted to those without hyperemesis gravidarum, preeclampsia or gestational diabetes mellitus. **Fig. S8.** Total GWG z-scores and RGWG with offspring NDCs (excluding those without IPI information). Spline models depicting the association of total GWG z-scores and RGWG with offspring NDCs, excluding those without IPI information. **Fig. S9.** RGWG-T2 and offspring NDCs (with additional adjustments and timing specification). Spline models depicting the association between RGWG-T2 and offspring NDCs, with additional adjustment for number of antenatal visits in the second trimester, and stratified by those who had the last weight measured < 25 and ≥ 25 weeks of gestation in the second trimester. **Fig. S10.** Impact of selection bias. The potential impact of selection bias in the association of total GWG z-scores, RGWG-T2, and RGWG-T3 with offspring NDCs by applying the inverse probability weight method.

**Additional file 2. **STROBE checklist. The STROBE checklist showing our study was reported according to the STROBE checklist for cohort studies.

## Data Availability

Data used for the current study were anonymized and obtained from Statistics Sweden and the National Board of Health and Welfare after ethical and legal assessment. Researchers interested in obtaining the data and replicating our results can make an inquiry through these data holders. For further information, see https://www.scb.se/en/services/guidance-for-researchers-and-universities/.

## Abbreviations

ADHD: Attention deficit/hyperactivity disorder
ASD: Autism spectrum disorder
BMI: Body mass index
CI: Confidence interval
GDM: Gestational diabetes mellitus
GWG: Gestational weight gain
HR: Hazard ratio
ICD: International Classification of Diseases
ID: Intellectual disability
IQR: Interquartile range
NDD: Neurodevelopmental disorders
RGWG-T2: Rate of gestational weight gain in the second trimester
RGWG-T3: Rate of gestational weight gain in the third trimester
