# Supplementary material for: Rates of maternal weight gain over the course of pregnancy and offspring risk of neurodevelopmental disorders
Source: BMC Med. 2023 Mar 23;21:108. doi: 10.1186/s12916-023-02799-6 (PMC10035205; doi:10.1186/s12916-023-02799-6)
Supplement: Supplementary file 1 — Additional file 1: Table S1. Included and excluded individuals. The characteristics of the included and excluded population in our study. Table S2. Diagnostic codes. The codes for identifying exposures, outcomes, and covariates. Table S3. Study cohort description by RGWG category. The characteristics of the study sample according to different categories of RGWG. Table S4. Total GWG category and offspring risk of NDCs in full cohort. The association between the 3-category total GWG and offspring risks of autism, ADHD and ID, and the mutuality exclusive diagnoses of these three NDCs. Table S5. RGWG category and offspring NDCs in full cohort. The association between the 3-catgory total RGWG and offspring risks of autism, ADHD and ID, and the mutuality exclusive diagnoses of these three NDCs. Table S6. RGWG in second and third trimester together with offspring NDCs in full cohort. The association between RGWG in second and third trimester in combination and offspring risks of NDCs. Fig. S1. Sample derivation and outcome description. A description of sample derivation and NDC co-occurrence in offspring. Fig. S2. Directed acyclic graph. A directed acyclic graph describing how potential confounders influence the exposures and outcomes. Fig. S3. Total GWG (kg) and offspring NDCs. Spline models depicting the association between GWG (kg) and offspring NDCs. Fig. S4. Visualization for the change of hazard ratios over time. We observed evidence in the cox regression models that the hazard ratios may be time varying for the association between excessive RGWG-T2 and any ADHD, and the association between extremely excessive RGWG-T3 and any ADHD. We therefore visualized the hazard ratios over time by using flexible parametric survival models for non-linear time-dependent effects. Fig. S5. Total GWG z-scores and offspring NDCs (sex stratification). Spline models depicting the association between total GWG z-scores and offspring NDCs, stratified by child’s sex. Fig. S6. Total GWG z-s [file 12916_2023_2799_MOESM1_ESM.docx]

# **Additional file 1**

Table S1. A comparison of individuals included in the final study sample to those excluded from the study sample

Table S2. Diagnostic codes and register databases used to ascertain diagnoses within the Stockholm Youth Cohort (SYC)

Table S3. Characteristics of the study cohort by rate of gestational weight gain category

Table S4. Association between gestational weight gain and offspring risk of neurodevelopment disorders in full cohort

Table S5. Association between rate of gestational weight gain at different stages of pregnancy and offspring risk of neurodevelopment disorders in full cohort

Table S6. Association of rate of gestational weight gain in second and third trimester with offspring risk of neurodevelopment disorders in full cohort

Fig S1. Selection of the study population and characterization of outcomes in the study cohort

Fig S2. Directed acyclic graph

Fig S3. Offspring risk for neurodevelopment disorders with respect to gestational weight gain (GWG) during pregnancy

Fig S4. Visualization for the change of hazard ratios over time.

Fig S5. Offspring risk for neurodevelopment disorders with respect to z score of gestational weight gain (GWG z-score) during pregnancy, stratified by sex.

Fig S6. Offspring risk for neurodevelopment disorders with respect to z score of gestational weight gain (GWG z-score) and rate of gestational weight gain during second (RGWG-T2) and third trimester (RGWG-T3), restricted to Nordic born.

Fig S7. Offspring risk for neurodevelopment disorders with respect to rate of gestational weight gain during second and third trimester (RGWG-T2/RGWG-T3), restricted to non-hyperemesis gravidarum, non-preeclampsia or non-gestational diabetes mellitus.

Fig S8. Offspring risk for neurodevelopment disorders with respect to total GWG z-scores and rates of gestational weight gain during second and third trimester (RGWG-T2/RGWG-T3), excluding those without IPI information.

Fig S9. Offspring risk for neurodevelopment disorders with respect to rate of gestational weight gain during second trimester (RGWG-T2), with additional adjustment for number of antenatal visits in the second trimester, with last weight measured < 25 and ≥25 weeks of gestation.

Fig S10. The potential impact of selection bias in the association of total GWG z-scores, RGWG-T2, and RGWG-T3 with NDDs in offspring.

## **Table S1.** **A comparison of individuals included in the final study sample to those excluded from the study sample**

|  | **Excluded in cohort** | **Included in cohort** |
| --- | --- | --- |
| **N** | 30215 | 57822 |
| **NDD diagnosis, %** |  |  |
| Any NDDs | 1328 (4.4%) | 2207 (3.8%) |
| Any ASD | 636 (2.1%) | 1120 (1.9%) |
| Any ADHD | 761 (2.5%) | 1354 (2.3%) |
| Any ID | 310 (1.0%) | 270 (0.5%) |
| ASD only | 308 (1.0%) | 650 (1.1%) |
| ADHD only | 522 (1.7%) | 961 (1.7%) |
| ASD+ADHD | 188 (0.6%) | 326 (0.6%) |
| ASD+ID | 140 (0.5%) | 144 (0.2%) |
| ID without ASD | 170 (0.6%) | 126 (0.2%) |
| **Child’s sex, %** |  |  |
| Male | 15730 (52.1%) | 29581 (51.2%) |
| Female | 14485 (47.9%) | 28241 (48.8%) |
| **Maternal age at birth (years), %** |  |  |
| < 25 | 3091 (10.2%) | 5811 (10.0%) |
| 25-29 | 7008 (23.2%) | 14586 (25.2%) |
| 30-34 | 11743 (38.9%) | 21763 (37.6%) |
| 35-39 | 6799 (22.5%) | 13014 (22.5%) |
| ≥40 | 1574 (5.2%) | 2648 (4.6%) |
| **Maternal birth region, %** |  |  |
| Nordic | 21516 (71.2%) | 42947 (74.3%) |
| Europe | 2052 (6.8%) | 3431 (5.9%) |
| Africa | 2053 (6.8%) | 2523 (4.4%) |
| Asia | 3650 (12.1%) | 7338 (12.7%) |
| Other | 940 (3.1%) | 1578 (2.7%) |
| Missing | 4 (<1%) | 5 (<1%) |
| **Paternal birth region, %** |  |  |
| Nordic | 20707 (68.5%) | 42021 (72.7%) |
| Europe | 2041 (6.8%) | 3394 (5.9%) |
| Africa | 2120 (7.0%) | 2795 (4.8%) |
| Asia | 3432 (11.4%) | 7051 (12.2%) |
| Other | 1113 (3.7%) | 1890 (3.3%) |
| Missing | 802 (2.7%) | 671 (1.2%) |
| **Maternal education level, %** |  |  |
| Pre-highschool | 3633 (12.0%) | 5897 (10.2%) |
| High school | 8975 (29.7%) | 19152 (33.1%) |
| Post-high school | 17195 (56.9%) | 32490 (56.2%) |
| Missing | 412 (1.4%) | 283 (0.5%) |
| **Parental disposable income at birth, %** |  |  |
| First (lowest) | 3713 (12.3%) | 4565 (7.9%) |
| Second | 5439 (18.0%) | 9889 (17.1%) |
| Third | 4511 (14.9%) | 9633 (16.7%) |
| Fourth | 5279 (17.5%) | 11883 (20.6%) |
| Fifth (highest) | 11114 (36.8%) | 21768 (37.6%) |
| Missing | 159 (0.5%) | 84 (0.1%) |
| **Interpregnancy interval (years), %** |  |  |
| First born | 13939 (46.1%) | 26811 (46.4%) |
| < 1 | 2468 (8.2%) | 4146 (7.2%) |
| 1-2 | 4557 (15.1%) | 9069 (15.7%) |
| 2-5 | 5118 (16.9%) | 10788 (18.7%) |
| 5-10 | 1355 (4.5%) | 3109 (5.4%) |
| >10 | 418 (1.4%) | 884 (1.5%) |
| Missing | 2360 (7.8%) | 3015 (5.2%) |
| **Maternal psychiatric history, %** | 3040 (10.1%) | 6050 (10.5%) |
| **Maternal smoking during pregnancy, %** |  |  |
| No | 27472 (90.9%) | 54220 (93.8%) |
| Yes | 1597 (5.3%) | 3067 (5.3%) |
| Missing | 1146 (3.8%) | 535 (0.9%) |
| **Maternal BMI at first antenatal visit, %** |  |  |
| Normal (18.5-25) | 18967 (62.8%) | 38975 (67.4%) |
| Underweight (>18.5) | 783 (2.6%) | 1765 (3.1%) |
| Overweight (25-30) | 6480 (21.4%) | 12264 (21.2%) |
| Obese (>30) | 2554 (8.5%) | 4818 (8.3%) |
| Missing | 1431 (4.7%) | 0 (0.0%) |

Table S2. Diagnostic codes and register databases used to ascertain diagnoses within the Stockholm Youth Cohort (SYC).

| **Coding system** | **Autism Spectrum Disorders (ASD)** | **Attention Deficit/Hyperactivity Disorder (ADHD)** | **Intellectual Disability (ID)** | **Congenital malformations, deformations, and chromosomal abnormalities** | ***Disorders of amino acid metabolism*** | ***Maternal psychiatric history*** |
| --- | --- | --- | --- | --- | --- | --- |
| ICD-10^a,b,c^ | F84 | F90 | F70-F79  F70: Mild (70>IQ≥50)  F71: Moderate (50>IQ≥35)  F72: Severe (35>IQ≥20)  F73: Profound (IQ<20)  F78/F79: Other/unspecified | Q90-99 (all)  Q85.0  Q85.1 | E70-E72 | F-chapter |
| Other |  | Prescription Drug Register^d^: methylphenidate [N06BA04] or atomoxetin [N06BA09] |  |  |  |  |

**Note:**

1. The National Patient Register (NPR): including inpatient care from 1973, outpatient physician visits in specialist care from 1997.
2. Stockholm Clinical Database for Child and Adolescent Psychiatry.
3. VAL register: Stockholm County health care databases including in- and outpatient care, regardless of specialty/primary health care.
4. The Prescription Drug Register (PDR) contains data on medications dispensed to the entire population in Sweden since 1 July 2005. Receipt of a prescription for ADHD medications is a useful proxy for an ADHD diagnosis, as Swedish medical guidelines mandate that ADHD medications should only be prescribed by a psychiatric specialist and after other (non-pharmacological) interventions have failed.

Abbreviations: ADHD = Attention-Deficit/Hyperactivity Disorder; ASD = Autism Spectrum Disorders; ICD = International Classification of Diseases; ID = Intellectual Disability; IQ = Intelligence Quotient

## **Table S3. Characteristics of the study cohort by total gestational weight gain category.**

| **Characteristic** | **GWG** | | |
| --- | --- | --- | --- |
|  | **Optimal** | **Insufficient** | **Excessive** |
| **N** | 23367 | 15583 | 18872 |
| **No. of measurements, median (IQR)** |  |  |  |
| 1^st^ trimester | 1.0 (1.0, 1.0) | 1.0 (1.0, 1.0) | 1.0 (1.0, 1.0) |
| 2^nd^ trimester | 1.0 (1.0, 2.0) | 1.0 (1.0, 2.0) | 1.0 (1.0, 2.0) |
| 3^rd^ trimester | 2.0 (1.0, 4.0) | 2.0 (1.0, 4.0) | 3.0 (2.0, 4.0) |
| **Gestational week at the first weight measurement, median (IQR)** |  |  |  |
| 1^st^ trimester | 9.3 (8.0, 10.4) | 9.3 (8.0, 10.7) | 9.0 (7.9, 10.3) |
| 2^nd^ trimester | 23.0 (19.9, 24.6) | 23.1 (20.0, 24.6) | 22.7 (19.7, 24.6) |
| 3^rd^ trimester | 31.0 (29.3, 35.0) | 30.9 (29.1, 34.3) | 30.7 (29.1, 34.4) |
| **Gestational week at the last weight measurement, median (IQR)** |  |  |  |
| 1^st^ trimester | 9.4 (8.1, 10.7) | 9.4 (8.3, 10.9) | 9.3 (8.0, 10.6) |
| 2^nd^ trimester | 25.0 (24.0, 26.0) | 24.9 (24.0, 26.0) | 25.0 (24.0, 26.1) |
| 3^rd^ trimester | 37.1 (36.0, 38.3) | 36.7 (35.3, 37.9) | 37.4 (36.4, 38.7) |
| **GWG (kg), mean (SD)** | 12.7 (2.6) | 8.5 (2.9) | 17.5 (3.9) |
| **RGWG-T2 (kg/week), mean (SD)** | 0.6 (0.2) | 0.4 (0.2) | 0.8 (0.3) |
| **RGWG-T2, %** |  |  |  |
| Optimal | 4337 (18.6%) | 5591 (35.9%) | 552 (2.9%) |
| Insufficient | 1654 (7.1%) | 5728 (36.8%) | 265 (1.4%) |
| Excessive | 17376 (74.4%) | 4264 (27.4%) | 18055 (95.7%) |
| **RGWG-T3, kg/week, mean (SD)** | 0.5 (0.2) | 0.3 (0.2) | 0.7 (0.2) |
| **RGWG-T3, %** |  |  |  |
| Optimal | 7086 (30.3%) | 5496 (35.3%) | 944 (5.0%) |
| Insufficient | 2482 (10.6%) | 7432 (47.7%) | 249 (1.3%) |
| Excessive | 13799 (59.1%) | 2655 (17.0%) | 17679 (93.7%) |
| **Child’s sex, %** |  |  |  |
| Male | 11940 (51.1%) | 7649 (49.1%) | 9992 (52.9%) |
| Female | 11427 (48.9%) | 7934 (50.9%) | 8880 (47.1%) |
| **Maternal BMI at first antenatal visit (kg/m^2^), %** |  |  |  |
| Normal (18.5-25) | 17505 (74.9%) | 12899 (82.8%) | 8571 (45.4%) |
| Underweight (>18.5) | 707 (3.0%) | 882 (5.7%) | 176 (0.9%) |
| Overweight (25-30) | 3633 (15.5%) | 1111 (7.1%) | 7520 (39.8%) |
| Obese (>30) | 1522 (6.5%) | 691 (4.4%) | 2605 (13.8%) |
| **Maternal age at birth (years), %** |  |  |  |
| < 25 | 2061 (8.8%) | 1455 (9.3%) | 2295 (12.2%) |
| 25-29 | 5771 (24.7%) | 3556 (22.8%) | 5259 (27.9%) |
| 30-34 | 9049 (38.7%) | 5984 (38.4%) | 6730 (35.7%) |
| 35-39 | 5428 (23.2%) | 3781 (24.3%) | 3805 (20.2%) |
| ≥40 | 1058 (4.5%) | 807 (5.2%) | 783 (4.1%) |
| **Maternal smoking during pregnancy, %** |  |  |  |
| No | 22097 (94.6%) | 14720 (94.5%) | 17403 (92.2%) |
| Yes | 1082 (4.6%) | 767 (4.9%) | 1218 (6.5%) |
| Missing | 188 (0.8%) | 96 (0.6%) | 251 (1.3%) |
| **Maternal birth region, %** |  |  |  |
| Nordic | 17691 (75.7%) | 11567 (74.2%) | 13689 (72.5%) |
| Europe | 1330 (5.7%) | 823 (5.3%) | 1278 (6.8%) |
| Africa | 952 (4.1%) | 896 (5.7%) | 675 (3.6%) |
| Asia | 2794 (12.0%) | 1879 (12.1%) | 2665 (14.1%) |
| Other | 599 (2.6%) | 418 (2.7%) | 561 (3.0%) |
| Missing | 1 (<1%) | 0 (0.0%) | NA (<1%) |
| **Paternal birth region, %** |  |  |  |
| Nordic | 17354 (74.3%) | 11440 (73.4%) | 13227 (70.1%) |
| Europe | 1358 (5.8%) | 838 (5.4%) | 1198 (6.3%) |
| Africa | 1031 (4.4%) | 931 (6.0%) | 833 (4.4%) |
| Asia | 2656 (11.4%) | 1684 (10.8%) | 2711 (14.4%) |
| Other | 716 (3.1%) | 490 (3.1%) | 684 (3.6%) |
| Missing | 252 (1.1%) | 200 (1.3%) | 219 (1.2%) |
| **Maternal education level, %** |  |  |  |
| Pre-highschool | 2097 (9.0%) | 1558 (10.0%) | 2242 (11.9%) |
| High-school | 7408 (31.7%) | 4626 (29.7%) | 7118 (37.7%) |
| Post-high school | 13769 (58.9%) | 9294 (59.6%) | 9427 (50.0%) |
| Missing | 93 (0.4%) | 105 (0.7%) | 85 (0.5%) |
| **Household income quintiles at birth, %** |  |  |  |
| First (lowest) | 1702 (7.3%) | 1319 (8.5%) | 1544 (8.2%) |
| Second | 3747 (16.0%) | 2648 (17.0%) | 3494 (18.5%) |
| Third | 3714 (15.9%) | 2470 (15.9%) | 3449 (18.3%) |
| Fourth | 4876 (20.9%) | 2958 (19.0%) | 4049 (21.5%) |
| Fifth (highest) | 9289 (39.8%) | 6167 (39.6%) | 6312 (33.4%) |
| Missing | 39 (0.2%) | 21 (0.1%) | 24 (0.1%) |
| **Interpregnancy interval (years) , %** |  |  |  |
| First born | 10615 (45.4%) | 6641 (42.6%) | 9555 (50.6%) |
| < 1 | 1697 (7.3%) | 1332 (8.5%) | 1117 (5.9%) |
| 1-2 | 3857 (16.5%) | 2744 (17.6%) | 2468 (13.1%) |
| 2-5 | 4431 (19.0%) | 3016 (19.4%) | 3341 (17.7%) |
| 5-10 | 1214 (5.2%) | 786 (5.0%) | 1109 (5.9%) |
| >10 | 344 (1.5%) | 228 (1.5%) | 312 (1.7%) |
| Missing | 1209 (5.2%) | 836 (5.4%) | 970 (5.1%) |
| **Maternal psychiatric history, %** | 2321 (9.9%) | 1642 (10.5%) | 2087 (11.1%) |

##

## **Table S4. Association between gestational weight gain and offspring risk of neurodevelopment disorders in full cohort**

| **GWG category ^a^** | **N cases** | **%** ^b^ | **Model 1** ^c^ | **Model 2** ^d^ | **P values** ^e^ |
| --- | --- | --- | --- | --- | --- |
| **Optimal** |  |  |  |  |  |
| Any NDDs | 846 | 3.62 | 1.00 (ref) | 1.00 (ref) | - |
| Any ASD | 448 | 1.92 | 1.00 (ref) | 1.00 (ref) | - |
| Any ADHD | 337 | 2.16 | 1.00 (ref) | 1.00 (ref) | - |
| Any ID | 65 | 0.42 | 1.00 (ref) | 1.00 (ref) | - |
| ASD only | 170 | 1.09 | 1.00 (ref) | 1.00 (ref) | - |
| ADHD only | 242 | 1.55 | 1.00 (ref) | 1.00 (ref) | - |
| ASD+ADHD | 77 | 0.49 | 1.00 (ref) | 1.00 (ref) | - |
| ASD+ID | 30 | 0.19 | 1.00 (ref) | 1.00 (ref) | - |
| ID without ASD | 35 | 0.22 | 1.00 (ref) | 1.00 (ref) | - |
| **Insufficient** |  |  |  |  |  |
| Any NDDs | 554 | 3.56 | 1.00 (0.90-1.11) | 0.98 (0.88-1.10) | 0.77 |
| Any ASD | 277 | 1.78 | 0.95 (0.82-1.10) | 0.94 (0.81-1.09) | 0.42 |
| Any ADHD | 334 | 2.19 | 1.02 (0.89-1.18) | 1.00 (0.87-1.15) | 0.95 |
| Any ID | 65 | 0.42 | 0.88 (0.65- 1.19) | 0.84 (0.62-1.14) | 0.25 |
| ASD only | 172 | 1.14 | 1.00 (0.83-1.22)* | 1.00 (0.83-1.22)* | 0.98 |
| ADHD only | 241 | 1.59 | 1.05 (0.89-1.23) | 1.03 (0.87-1.21) | 0.75 |
| ASD+ADHD | 77 | 0.51 | 0.96 (0.72-1.28) | 0.95 (0.72-1.27) | 0.73 |
| ASD+ID | 30 | 0.20 | 0.71 (0.46-1.09) | 0.67 (0.43-1.03) | 0.07 |
| ID without ASD | 36 | 0.24 | 1.12 (0.72-1.73) | 1.07 (0.69-1.66) | 0.76 |
| **Excessive** |  |  |  |  |  |
| Any NDDs | 805 | 4.27 | **1.15 (1.05-1.27)** | 1.04 (0.95-1.15) | 0.39 |
| Any ASD | 394 | 2.09 | 1.07 (0.94-1.23) | 0.99 (0.87-1.14) | 0.93 |
| Any ADHD | 515 | 2.73 | **1.24 (1.10-1.41)** | 1.10 (0.97-1.25) | 0.13 |
| Any ID | 93 | 0.49 | 1.02 (0.78-1.35) | 0.94 (0.72-1.24) | 0.68 |
| ASD only | 219 | 1.16 | 1.03 (0.86-1.23) | 0.97 (0.81-1.16) | 0.72 |
| ADHD only | 367 | 1.94 | **1.27 (1.09-1.47)** | 1.12 (0.96-1.30) | 0.14 |
| ASD+ADHD | 126 | 0.67 | 1.24 (0.97-1.58) | 1.10 (0.85-1.41) | 0.47 |
| ASD+ID | 49 | 0.26 | 0.92 (0.64-1.34) | 0.89 (0.61-1.29) | 0.53 |
| ID without ASD | 44 | 0.23 | 1.16 (0.77-1.75) | 1.02 (0.68-1.55) | 0.92 |

Abbreviations: GWG, gestational weight gain; NDDs, neurodevelopmental disorders; ASD, autism spectrum disorder; ADHD, attention-deficit/hyperactivity disorder; ID, intellectual disability

^a^ Total GWG were categorized based on IOM recommendations for each BMI category as optimal, insufficient, or excessive. For normal weight women, the optimal GWG was 11.5 - 16 kg, the insufficient GWG was <11.5 kg, the excessive GWG was >16 kg. For underweight women, the optimal GWG was 11.5 - 16 kg, the insufficient GWG was <11.5 kg, the excessive GWG was >16 kg. For overweight women, the optimal GWG was 7 - 11.5 kg, the insufficient GWG was <7 kg, the excessive GWG was >11.5 kg. For obese women, the optimal GWG was 5 - 9 kg, the insufficient GWG was <5 kg, the excessive GWG was >9 kg.

^b^ Calculated as the number of cases observed when following children from 2 years of age for mean [SD] of 5.4 [1.1] years, divided by the number of children at risk for developing the disorder.

^c^ Model 1: Cox regression model, clustered on maternal identifier, adjusted only for birth year and child’s sex. Results are displayed as the Hazard Ratio (95% Confidence Interval).

^d^ Model 2: Cox regression model, clustered on maternal identifier, adjusted for birth year, child’s sex, maternal age at birth, household income quintiles at birth, maternal education level, parental birth region, interpregnancy interval, maternal psychiatric history, and maternal smoking during pregnancy. Results are displayed as the Hazard Ratio (95% Confidence Interval).

^e^ P values for model 2.

* An interaction with time was observed for these categories, indicating that the HR changes over time.

## **Table S5. Association between** **rate of gestational weight gain at different stages of pregnancy and offspring risk of neurodevelopment disorders in full cohort.**

| **Simple category^a^** | **RGWG-T2** | | | |  |  | **RGWG-T3** | | | |  |
| --- | --- | --- | --- | --- | --- | --- | --- | --- | --- | --- | --- |
|  | **N cases** | **%** ^b^ | **Model 1 ^c^** | **Model 2 ^d^** | **P values ^e^** |  | **N cases** | **%** ^b^ | **Model 1 ^c^** | **Model 2 ^d^** | **P values ^e^** |
| **Optimal** |  |  |  |  |  |  |  |  |  |  |  |
| Any NDDs | 380 | 3.63 | 1.00 (ref) | 1.00 (ref) | - |  | 469 | 3.47 | 1.00 (ref) | 1.00 (ref) | - |
| Any ASD | 207 | 1.98 | 1.00 (ref) | 1.00 (ref) | - |  | 253 | 1.87 | 1.00 (ref) | 1.00 (ref) | - |
| Any ADHD | 217 | 2.07 | 1.00 (ref) | 1.00 (ref) | - |  | 278 | 2.06 | 1.00 (ref) | 1.00 (ref) | - |
| Any ID | 42 | 0.55 | 1.00 (ref) | 1.00 (ref) | - |  | 51 | 0.38 | 1.00 (ref) | 1.00 (ref) | - |
| ASD only | 134 | 1.28 | 1.00 (ref) | 1.00 (ref) | - |  | 155 | 1.15 | 1.00 (ref) | 1.00 (ref) | - |
| ADHD only | 159 | 1.52 | 1.00 (ref) | 1.00 (ref) | - |  | 195 | 1.44 | 1.00 (ref) | 1.00 (ref) | - |
| ASD+ADHD | 45 | 0.43 | 1.00 (ref) | 1.00 (ref) | - |  | 68 | 0.50 | 1.00 (ref) | 1.00 (ref) | - |
| ASD+ID | 28 | 0.27 | 1.00 (ref) | 1.00 (ref) | - |  | 30 | 0.22 | 1.00 (ref) | 1.00 (ref) | - |
| ID without ASD | 14 | 0.13 | 1.00 (ref) | 1.00 (ref) | - |  | 21 | 0.16 | 1.00 (ref) | 1.00 (ref) | - |
| **Insufficient** |  |  |  |  |  |  |  |  |  |  |  |
| Any NDDs | 343 | 4.49 | **1.26 (1.09-1.46)** | 1.14 (0.98-1.32) | 0.08 |  | 343 | 3.37 | 0.98 (0.85-1.12) | 0.94 (0.81-1.08) | 0.35 |
| Any ASD | 164 | 2.14 | 1.11 (0.90-1.36) | 1.04 (0.84-1.28) | 0.72 |  | 174 | 1.71 | 0.92 (0.76-1.12) | 0.89 (0.74-1.08) | 0.25 |
| Any ADHD | 230 | 3.01 | **1.48 (1.23-1.78)** | **1.30 (1.08-1.57)** | **0.006** |  | 218 | 2.15 | 1.05 (0.88-1.25) | 1.00 (0.83-1.19) | 0.98 |
| Any ID | 42 | 0.55 | 1.40 (0.91-2.14) | 1.20 (0.78-1.85) | 0.41 |  | 40 | 0.39 | 1.05 (0.69-1.58) | 0.93 (0.62-1.41) | 0.74 |
| ASD only | 81 | 1.06 | 0.85 (0.64-1.12) | 0.82 (0.62-1.08) | 0.16 |  | 95 | 0.93 | 0.82 (0.64-1.06) | 0.81 (0.63-1.04) | 0.10 |
| ADHD only | 158 | 2.07 | **1.39 (1.11-1.73)** | 1.21 (0.97-1.50) | 0.10 |  | 151 | 1.49 | 1.03 (0.83-1.28) | 0.98 (0.80-1.22) | 0.88 |
| ASD+ADHD | 62 | 0.81 | **1.92 (1.31-2.82)** | **1.75 (1.19-2.57)** | **0.004** |  | 57 | 0.56 | 1.13 (0.79-1.60) | 1.09 (0.77-1.55) | 0.63 |
| ASD+ID | 21 | 0.27 | 1.05 (0.60-1.85) | 0.92 (0.51-1.64) | 0.77 |  | 22 | 0.22 | 0.98 (0.57-1.71) | 0.88 (0.51-1.52) | 0.65 |
| ID without ASD | 21 | 0.27 | **2.08 (1.06-4.10)** | 1.77 (0.91-3.48) | 0.09 |  | 18 | 0.18 | 1.14 (0.61-2.13) | 1.01 (0.54-1.89) | 0.97 |
| **Excessive** |  |  |  |  |  |  |  |  |  |  |  |
| Any NDDs | 1,482 | 3.73 | 1.01 (0.90-1.13) | 0.97 (0.87-1.09) | 0.64 |  | 1,393 | 4.08 | **1.17 (1.05-1.29)** | 1.07 (0.96-1.19) | 0.24 |
| Any ASD | 748 | 1.88 | 0.93 (0.80-1.09) | 0.91 (0.78-1.06) | 0.22 |  | 692 | 2.03 | 1.07 (0.93-1.24) | 1.01 (0.87-1.16) | 0.95 |
| Any ADHD | 906 | 2.28 | 1.08 (0.93-1.25) | 1.03 (0.89-1.20) | 0.68 |  | 857 | 2.51 | **1.21 (1.06-1.39)** | 1.09 (0.95-1.25) | 0.24 |
| Any ID | 186 | 0.47 | 1.16 (0.83-1.62) | 1.13 (0.81-1.58) | 0.47 |  | 179 | 0.52 | **1.39 (1.02-1.90)** | 1.29 (0.95-1.77) | 0.11 |
| ASD only | 434 | 1.09 | 0.84 (0.69-1.02) | 0.82 (0.67-1.00) | **0.04** |  | 399 | 1.17 | 1.01 (0.84-1.22) | 0.96 (0.79-1.16) | 0.64 |
| ADHD only | 643 | 1.62 | 1.04 (0.88-1.24) | 1.00 (0.84-1.19)* | 0.98 |  | 614 | 1.80 | **1.24 (1.05-1.46)** | 1.10 (0.94-1.30) | 0.24 |
| ASD+ADHD | 219 | 0.55 | 1.25 (0.91-1.72) | 1.18 (0.86-1.63) | 0.31 |  | 201 | 0.59 | 1.16 (0.88-1.53) | 1.05 (0.80-1.39) | 0.73 |
| ASD+ID | 95 | 0.24 | 0.88 (0.58-1.35) | 0.88 (0.57-1.34) | 0.54 |  | 92 | 0.27 | 1.21 (0.80-1.82) | 1.17 (0.77-1.77) | 0.46 |
| ID without ASD | 91 | 0.23 | 1.71 (0.98-3.00) | 1.63 (0.93-2.86) | 0.09 |  | 87 | 0.25 | **1.65 (1.02-2.66)** | 1.48 (0.91-2.39) | 0.11 |

Abbreviations: RGWG, rate of gestational weight gain; C.I., Ref, reference; NDDs, neurodevelopmental disorders; ASD, autism spectrum disorder; ADHD, attention-deficit/hyperactivity disorder; ID, intellectual disability;

^a^ For normal weight women, the optimal rate of weight gain during the second and third trimesters was 0.35 - 0.50 kg/week, the insufficient rate was <0.35 kg/week, the excessive rate was >0.50 kg/week.

For underweight women, the optimal rate was 0.44 - 0.58 kg/week, the insufficient rate was <0.44 kg/week, the excessive rate was >0.58 kg/week.

For overweight women, the optimal rate was 0.23 - 0.33 kg/week, the insufficient rate was <0.23 kg/week, the excessive rate was >0.33 kg/week.

For obese women, the optimal rate was 0.17 - 0.27 kg/week, the insufficient rate was <0.17 kg/week, the excessive rate was >0.27 kg/week.

^b^ Calculated as the number of cases observed when following children from 2 years of age for mean [SD] of 5.4 [1.1] years, divided by the number of children at risk for developing the disorder.

^c^ Model 1: Cox regression model, clustered on maternal identifier, adjusted only for birth year and child’s sex. Results are displayed as the Hazard Ratio (95% Confidence Interval).

^d^ Model 2: Cox regression model, clustered on maternal identifier, adjusted for birth year, child’s sex, maternal age at birth, household income quintiles at birth, maternal education level, parental birth region, interpregnancy interval, maternal psychiatric history, and maternal smoking during pregnancy. Results are displayed as the Hazard Ratio (95% Confidence Interval).

^e^ P values for model 2.

* An interaction with time was observed for these categories, indicating that the HR changes over time.

## **Table S6. Association of rate of gestational weight gain in second and third trimester with offspring risk of neurodevelopment disorders in full cohort ^a^.**

|  | **N cases** | **%** ^b^ | **Model 1 ^c^** | **Model 2 ^d^** | **P values ^e^** |
| --- | --- | --- | --- | --- | --- |
| **Any NDDs** |  |  |  |  |  |
| Optimal RGWG-T2, optimal RGWG-T3 | 109 | 3.45 | 1.00 (ref) | 1.00 (ref) | - |
| Optimal RGWG-T2, insufficient RGWG-T3 | 80 | 3.25 | 0.96 (0.72-1.29) | 0.92 (0.69-1.22) | 0.56 |
| Optimal RGWG-T2, excessive RGWG-T3 | 191 | 3.93 | 1.16 (0.92-1.47) | 1.07 (0.84-1.35) | 0.60 |
| Insufficient RGWG-T2, optimal RGWG-T3 | 80 | 3.91 | 1.18 (0.88-1.57) | 1.07 (0.80-1.43) | 0.64 |
| Insufficient RGWG-T2, insufficient RGWG-T3 | 81 | 3.88 | 1.16 (0.87-1.55) | 0.97 (0.73-1.30) | 0.85 |
| Insufficient RGWG-T2, excessive RGWG-T3 | 182 | 5.18 | **1.54 (1.22-1.96)** | **1.31 (1.03-1.66)** | **0.03** |
| Excessive RGWG-T2, optimal RGWG-T3 | 280 | 3.36 | 0.98 (0.79-1.22) | 0.96 (0.77-1.19) | 0.69 |
| Excessive RGWG-T2, insufficient RGWG-T3 | 182 | 3.24 | 0.94 (0.74-1.19) | 0.90 (0.72-1.14) | 0.39 |
| Excessive RGWG-T2, excessive RGWG-T3 | 1,020 | 3.96 | 1.14 (0.93-1.38) | 1.01 (0.83-1.23) | 0.91 |
| **Any ASD** |  |  |  |  |  |
| Optimal RGWG-T2, optimal RGWG-T3 | 61 | 1.93 | 1.00 (ref) | 1.00 (ref) | - |
| Optimal RGWG-T2, insufficient RGWG-T3 | 49 | 1.99 | 1.05 (0.72-1.53) | 1.02 (0.70-1.48) | 0.94 |
| Optimal RGWG-T2, excessive RGWG-T3 | 97 | 1.99 | 1.05 (0.77-1.45) | 0.99 (0.72-1.36) | 0.96 |
| Insufficient RGWG-T2, optimal RGWG-T3 | 39 | 1.91 | 1.03 (0.69-1.54) | 0.97 (0.65-1.45) | 0.88 |
| Insufficient RGWG-T2, insufficient RGWG-T3 | 35 | 1.68 | 0.90 (0.60-1.37) | 0.80 (0.52-1.21) | 0.29 |
| Insufficient RGWG-T2, excessive RGWG-T3 | 90 | 2.56 | 1.36 (0.98-1.89) | 1.22 (0.88-1.69) | 0.23 |
| Excessive RGWG-T2, optimal RGWG-T3 | 153 | 1.84 | 0.95 (0.71-1.28) | 0.93 (0.69-1.26) | 0.65 |
| Excessive RGWG-T2, insufficient RGWG-T3 | 90 | 1.60 | 0.83 (0.60-1.15) | 0.80 (0.58-1.11) | 0.19 |
| Excessive RGWG-T2, excessive RGWG-T3 | 505 | 1.96 | 1.00 (0.77-1.31) | 0.92 (0.70-1.20) | 0.53 |
| **Any ADHD** |  |  |  |  |  |
| Optimal RGWG-T2, optimal RGWG-T3 | 57 | 1.81 | 1.00 (ref) | 1.00 (ref) | - |
| Optimal RGWG-T2, insufficient RGWG-T3 | 48 | 1.95 | 1.11 (0.75-1.62) | 1.03 (0.71-1.51) | 0.87 |
| Optimal RGWG-T2, excessive RGWG-T3 | 112 | 2.30 | 1.31 (0.95-1.80) | 1.16 (0.85-1.60) | 0.35 |
| Insufficient RGWG-T2, optimal RGWG-T3 | 53 | 2.59 | **1.48 (1.02-2.15)** | 1.29 (0.89-1.88) | 0.18 |
| Insufficient RGWG-T2, insufficient RGWG-T3 | 59 | 2.83 | **1.61 (1.11-2.33)** | 1.29 (0.89-1.87) | 0.18 |
| Insufficient RGWG-T2, excessive RGWG-T3 | 118 | 3.36 | **1.93 (1.41-2.65)** | **1.55 (1.13-2.13)** | **0.007** |
| Excessive RGWG-T2, optimal RGWG-T3 | 168 | 2.02 | 1.12 (0.83-1.52) | 1.08 (0.80-1.46) | 0.60 |
| Excessive RGWG-T2, insufficient RGWG-T3 | 11 | 1.98 | 1.09 (0.80-1.50) | 1.04 (0.76-1.43) | 0.80 |
| Excessive RGWG-T2, excessive RGWG-T3 | 627 | 2.43 | **1.33 (1.02-1.75)** | 1.15 (0.88-1.50) | 0.32 |
| **Any ID** |  |  |  |  |  |
| Optimal RGWG-T2, optimal RGWG-T3 | 8 | 0.25 | 1.00 (ref) | 1.00 (ref) | - |
| Optimal RGWG-T2, insufficient RGWG-T3 | 9 | 0.37 | 1.46 (0.56-3.77) | 1.30 (0.50-3.36) | 0.59 |
| Optimal RGWG-T2, excessive RGWG-T3 | 25 | 0.51 | 2.05 (0.93-4.55)* | 1.91 (0.86-4.24)* | 0.11 |
| Insufficient RGWG-T2, optimal RGWG-T3 | 6 | 0.29 | 1.19 (0.41-3.43) | 1.07 (0.37-3.06) | 0.91 |
| Insufficient RGWG-T2, insufficient RGWG-T3 | 9 | 0.4 | 1.74 (0.67-4.52) | 1.28 (0.49-3.36) | 0.61 |
| Insufficient RGWG-T2, excessive RGWG-T3 | 27 | 0.77 | **3.12 (1.41-6.85)** | **2.53 (1.15-5.55)** | **0.02** |
| Excessive RGWG-T2, optimal RGWG-T3 | 37 | 0.44 | 1.75 (0.82-3.76) | 1.70 (0.79-3.66) | 0.17 |
| Excessive RGWG-T2, insufficient RGWG-T3 | 22 | 0.39 | 1.54 (0.69-3.46) | 1.40 (0.62-3.13) | 0.42 |
| Excessive RGWG-T2, excessive RGWG-T3 | 127 | 0.49 | 1.94 (0.95-3.96) | 1.76 (0.86-3.60) | 0.12 |

Abbreviations: RGWG, rate of gestational weight gain; NDDs, neurodevelopmental disorders; ASD, autism spectrum disorder; ADHD, attention-deficit/hyperactivity disorder; ID, intellectual disability; GEE, generalized estimating equation.

^a^ Rate of GWG in second and third trimester were categorized based on IOM recommendations for each BMI category as optimal, insufficient, or excessive. Optimal ranges for underweight: 0.44-0.58 kg/week; normal BMI 0.35-0.50 kg/week; overweight 0.23-0.33 kg/week; obese: 0.17-0.27 kg/week.

^b^ Calculated as the number of cases observed when following children from 2 years of age for mean [SD] of 5.4 [1.1] years, divided by the number of children at risk for developing the disorder.

^c^ Model 1: Cox regression model, clustered on maternal identifier, adjusted only for birth year and child’s sex. Results are displayed as the Hazard Ratio (95% Confidence Interval).

^d^ Model 2: Cox regression model, clustered on maternal identifier, adjusted for birth year, child’s sex, maternal age at birth, household income quintiles at birth, maternal education level, parental birth region, interpregnancy interval, maternal psychiatric history, and maternal smoking during pregnancy. Results are displayed as the Hazard Ratio (95% Confidence Interval).

^e^ P values for model 2.

* An interaction with time was observed for these categories, indicating that the HR changes over time.

## **Fig S1. Selection of the study population and characterization of outcomes in the study cohort.**


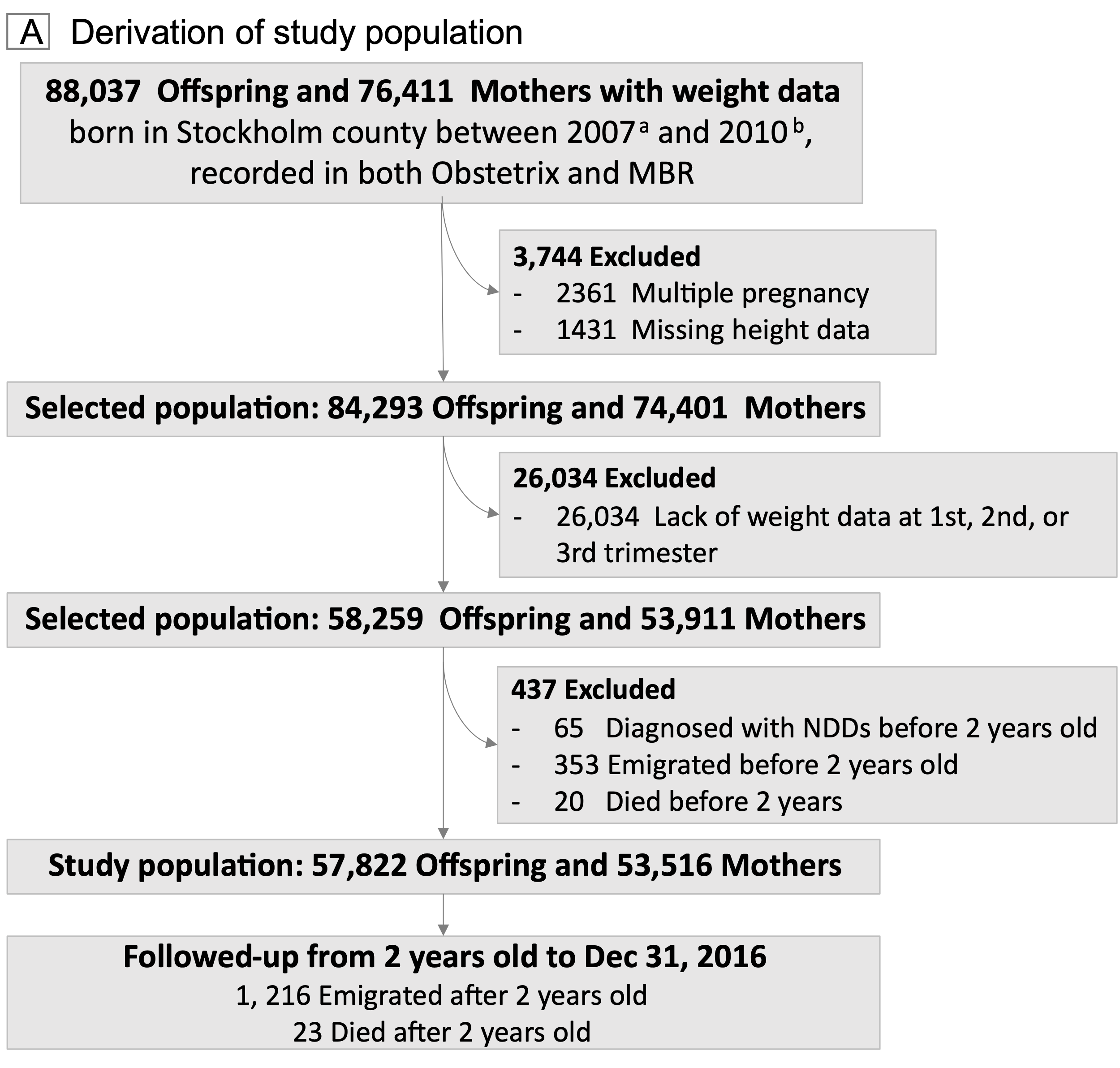


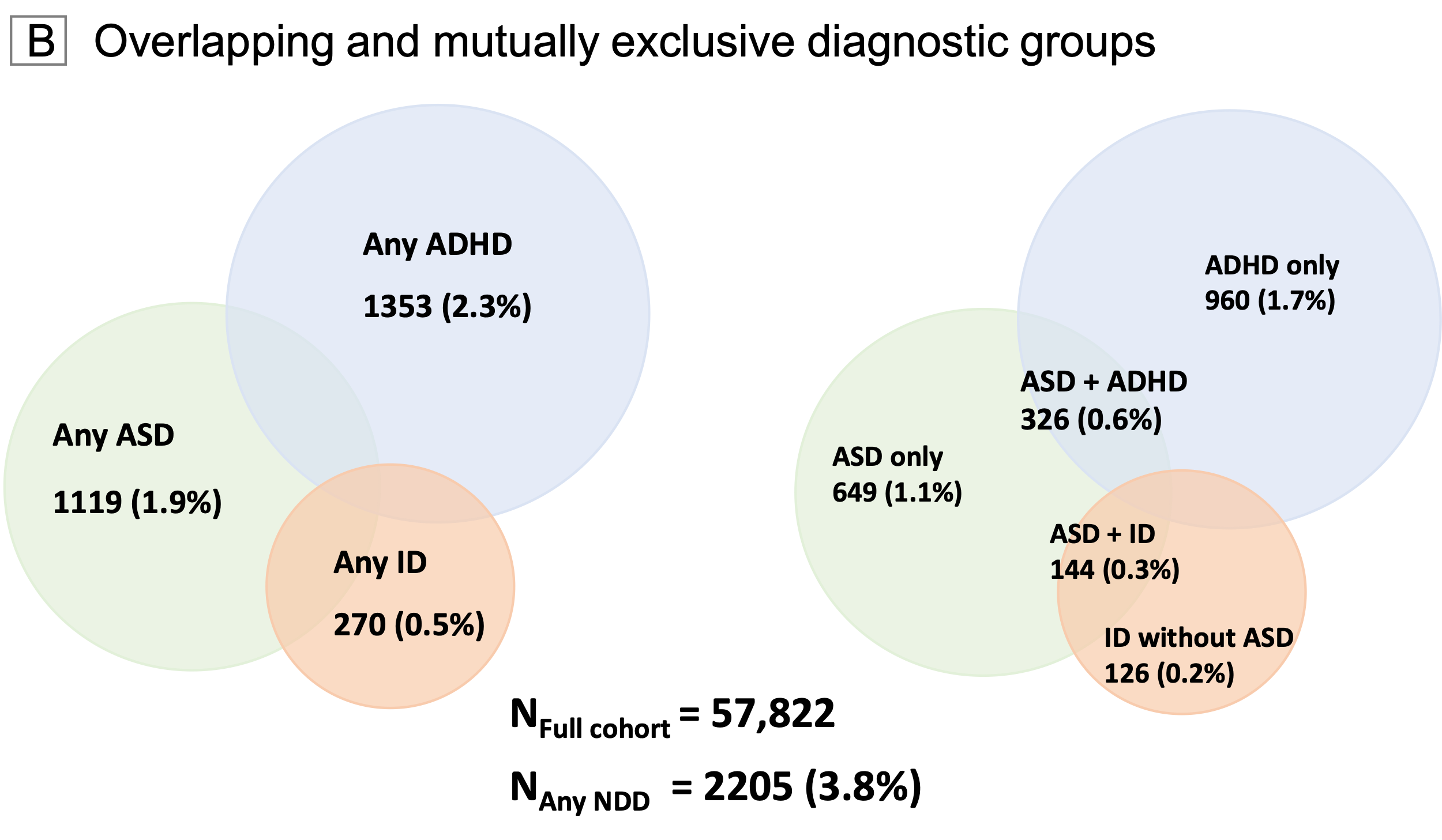


a Children born before 2007 were excluded from the study population since Obstetrix data collection began in 2007.

b Children born in 2011 were excluded from the study population since Medical Birth Register (MBR) data were not yet available from 2011.

**Fig S2. Directed acyclic graph**

**
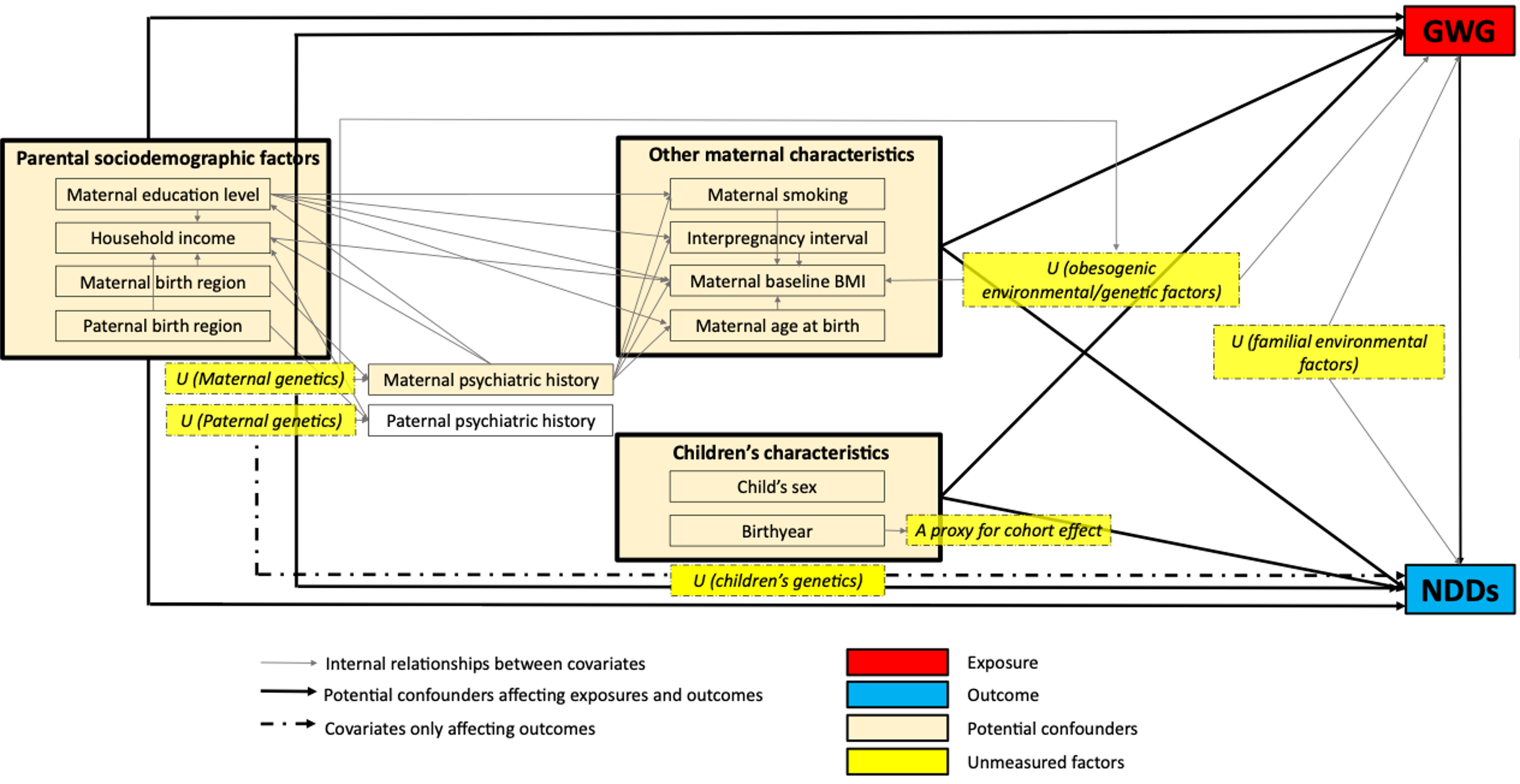
**

(Source: http://www.dagitty.net/)

The included confounders were child’s sex, birth year, household income quintiles at birth, maternal age at birth, maternal education level, parental birth region (i.e., maternal and paternal birth region), interpregnancy interval (IPI), maternal smoking during pregnancy, and maternal psychiatric history prior to the birth of the child.

**Fig S3. Offspring risk for neurodevelopment disorders with respect to total gestational weight gain (GWG, in kilograms) during pregnancy**. Histograms illustrate the distribution of GWG for those included in each analysis. Adjusted estimates are shown for any NDDs, ASD, ADHD, and ID. The curved solid black line represents the Hazard ratio (HR) calculated through restricted cubic splines models with 3 knots. The grey bands represent the 95% CI. A reference line is included for an HR of 1.00. The model was adjusted for birth year, sex, maternal age, household income quintile, maternal education level, parental birth region, interpregnancy interval, maternal psychiatric history, maternal smoking during pregnancy, and maternal BMI at first antenatal visit (only in the full cohort analysis). Note that the y-scale differs for ID compared to the other outcomes.
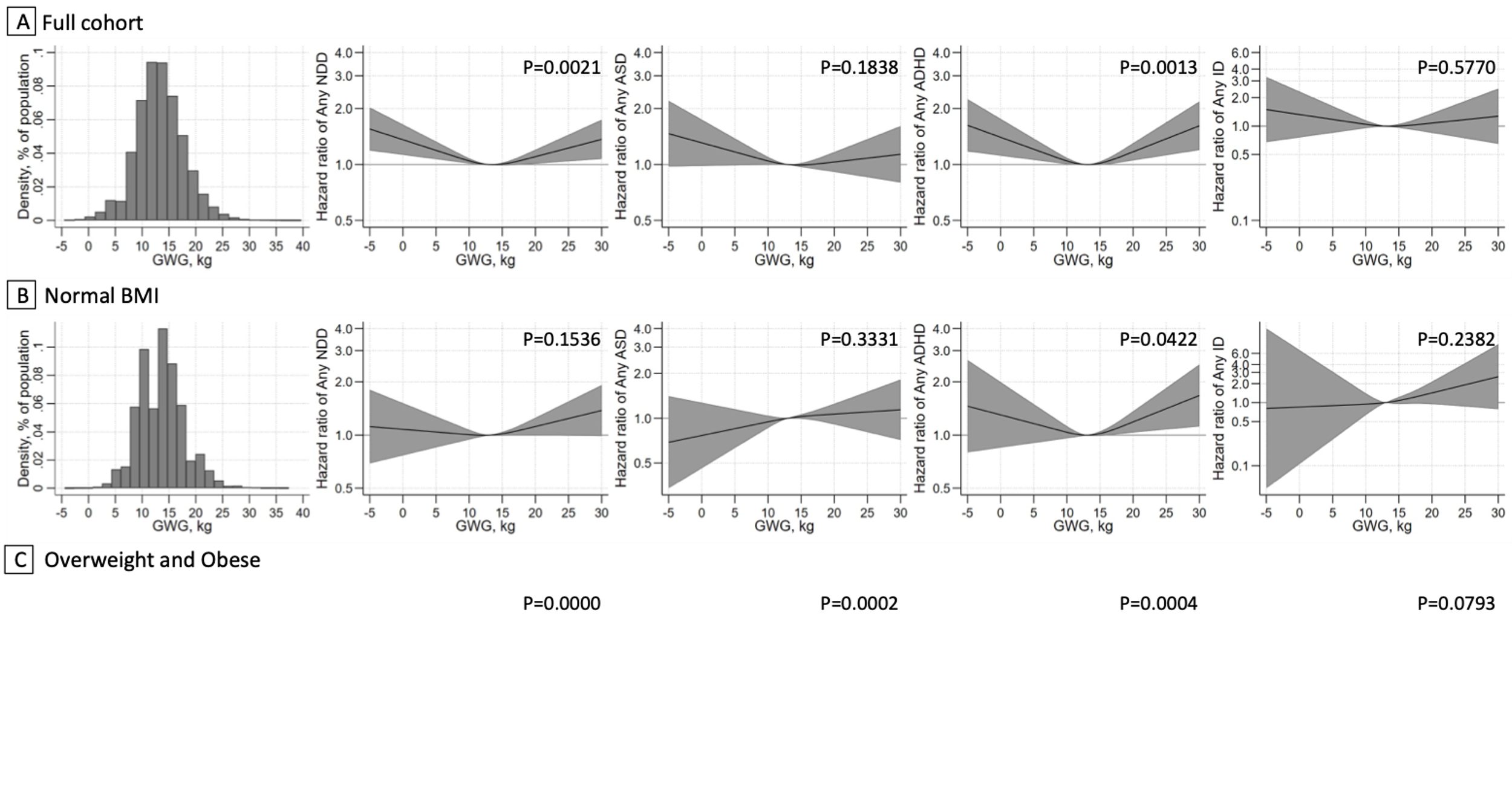


**Fig S4. Visualization for the change of hazard ratios over time.** We observed evidence in the cox regression models that the hazard ratios may be time varying for the association between excessive RGWG-T2 and any ADHD, and the association between extremely excessive RGWG-T3 and any ADHD. We therefore visualized the hazard ratios over time by using flexible parametric survival models with 4 degrees of freedom (knots were placed at 25^th^, 50^th^, and 75^th^ centiles of the distribution of the uncensored log survival times) for non-linear time-dependent effects. P values for the time-varying effects are shown for a Wald test with a null hypothesis that all time interaction terms in the splines were jointly equal to zero. The model was adjusted for birth year, child’s sex, maternal age at birth, household income quintiles at birth, maternal education level, parental birth region, interpregnancy interval, maternal psychiatric history, maternal smoking during pregnancy, and maternal BMI at first antenatal visit.


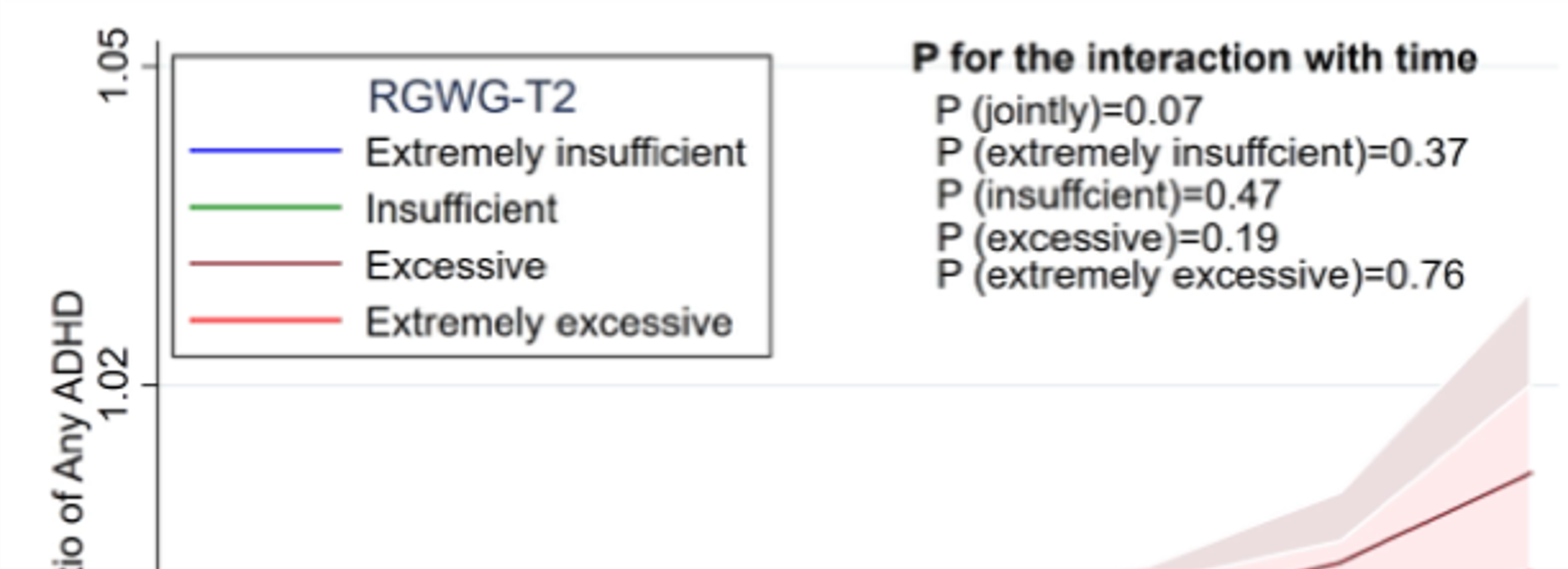


## **Fig S5. Offspring risk for neurodevelopment disorders with respect to z score of total gestational weight gain (GWG z-score) during pregnancy, stratified by sex.** Histograms illustrate the distribution of GWG z-score for those included in each analysis. Adjusted estimates are shown for any NDDs, ASD, ADHD, and ID. The curved solid black line represents the Hazard ratio (HR) calculated through restricted cubic splines models with 3 knots. The grey bands represent the 95% CI. A reference line is included for an HR of 1.00. The model was adjusted for birth year, maternal age at birth, household income quintiles at birth, maternal education level, parental birth region, interpregnancy interval, maternal psychiatric history, maternal smoking during pregnancy, and maternal BMI at first antenatal visit. Note that the y-scale differs for ID compared to the other outcomes.


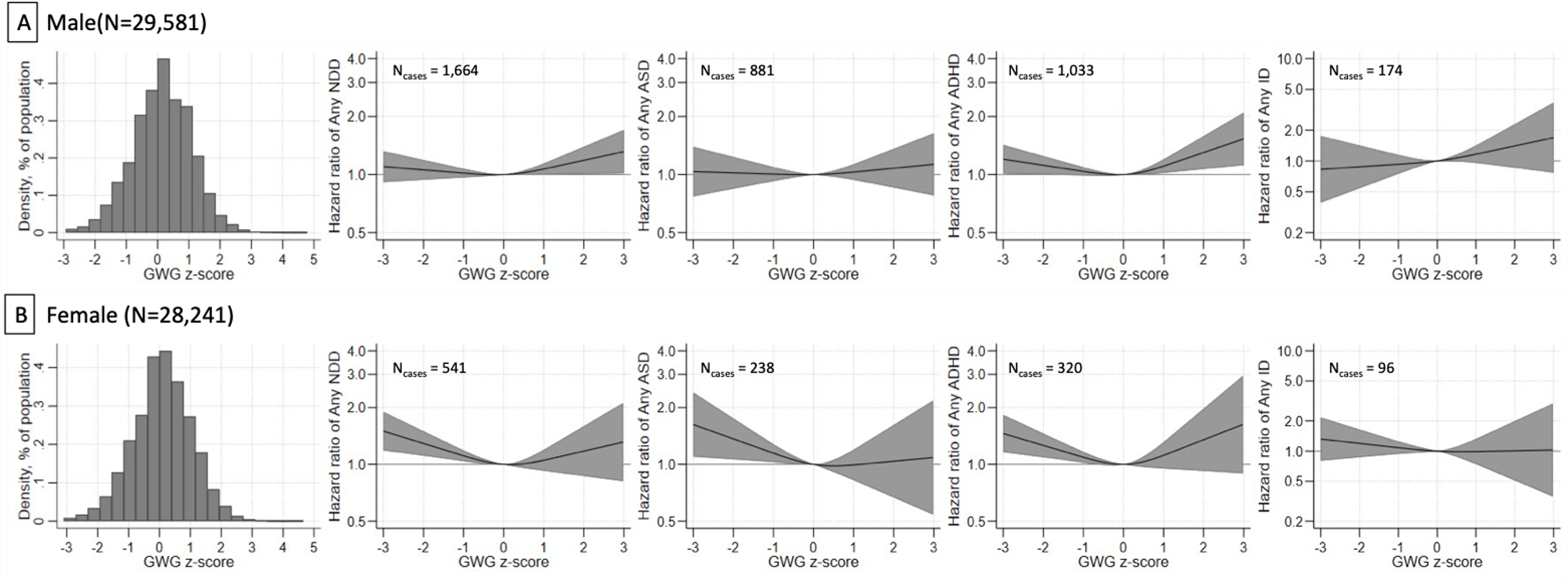


## **Fig S6. Offspring risk for neurodevelopment disorders with respect to z score of gestational weight gain (GWG z-score) and rate of gestational weight gain during second (RGWG-T2) and third trimester (RGWG-T3), restricted to Nordic born (N=42,947).** Histograms illustrate the distribution of GWG z-score for those included in each analysis. Adjusted estimates are shown for any NDDs, ASD, ADHD, and ID The curved solid black line represents the Hazard ratio (HR) calculated through restricted cubic splines models with 3 knots. The grey bands represent the 95% CI. A reference line is included for an HR of 1.00. The model was adjusted for birth year, child’s sex, maternal age at birth, household income quintiles at birth, maternal education level, paternal birth region, interpregnancy interval, maternal psychiatric history, maternal smoking during pregnancy, and maternal BMI at first antenatal visit. Note that the y-scale differs for ID compared to the other outcomes.

##
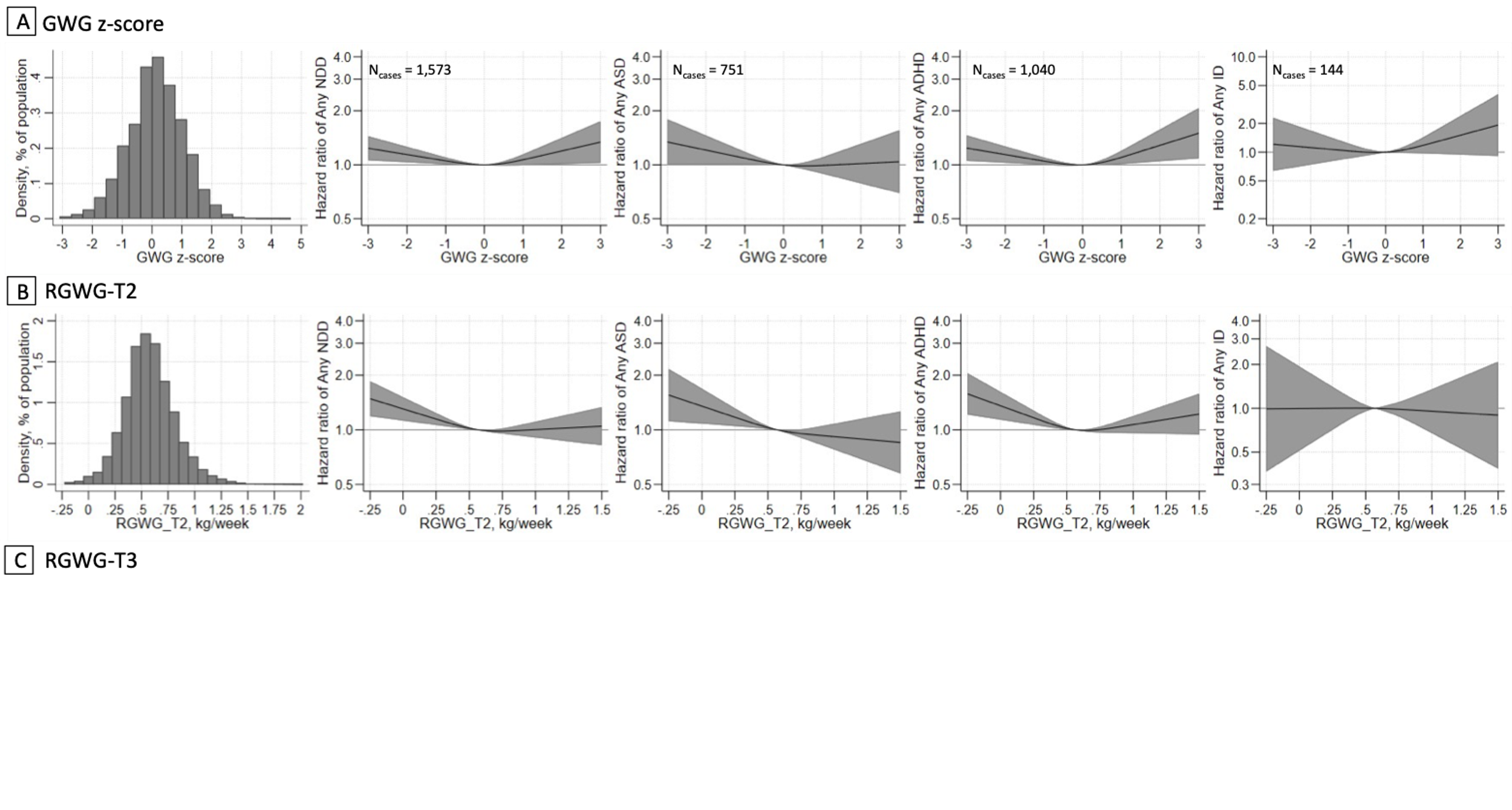


## **Fig S7. Offspring risk for neurodevelopment disorders with respect to rate of gestational weight gain during second and third trimester (RGWG-T2/RGWG-T3), restricted to non-hyperemesis gravidarum, non-preeclampsia or non-gestational diabetes mellitus.** Histograms illustrate the distribution of RGWG-T3 for those included in each analysis. Adjusted estimates are shown for any NDDs, ASD, ADHD, and ID. The curved solid black line represents the Hazard ratio (HR) calculated through restricted cubic splines models with 3 knots. The grey bands represent the 95% CI. A reference line is included for an HR of 1.00. The model was adjusted for birth year, sex, maternal age, household income quintile, maternal education level, parental birth region, interpregnancy interval, maternal psychiatric history, maternal smoking during pregnancy, and maternal BMI at first antenatal visit. Note that the y-scale differs for ID compared to the other outcomes.


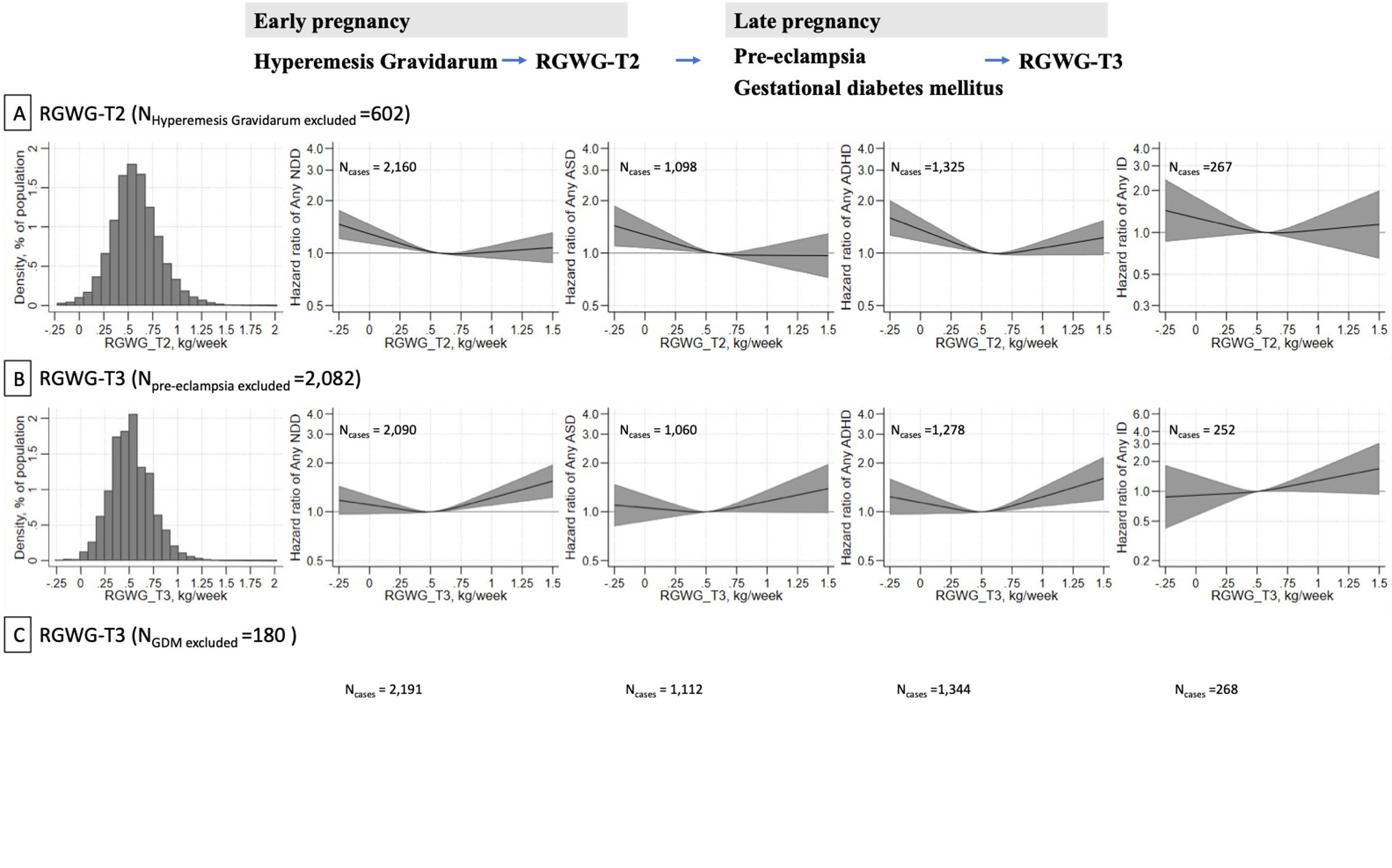


**Fig S8.** **Offspring risk for neurodevelopment disorders with respect to total GWG z-scores and rates of gestational weight gain during second and third trimester (RGWG-T2/RGWG-T3), excluding those without IPI information (N_excluded_ =3015, 5.2%).** Histograms illustrate the distribution of GWG z-scores, RGWG-T2, and -T3 for those included in each analysis. Adjusted estimates are shown for any NDDs, ASD, ADHD, and ID. The curved solid black line represents the Hazard ratio (HR) calculated through restricted cubic splines models with 3 knots. The grey bands represent the 95% CI. A reference line is included for an HR of 1.00. The model was adjusted for birth year, sex, maternal age, household income quintile, maternal education level, parental birth region, interpregnancy interval, maternal psychiatric history, maternal smoking during pregnancy, and maternal BMI at first antenatal visit. Note that the y-scale differs for ID compared to the other outcomes.


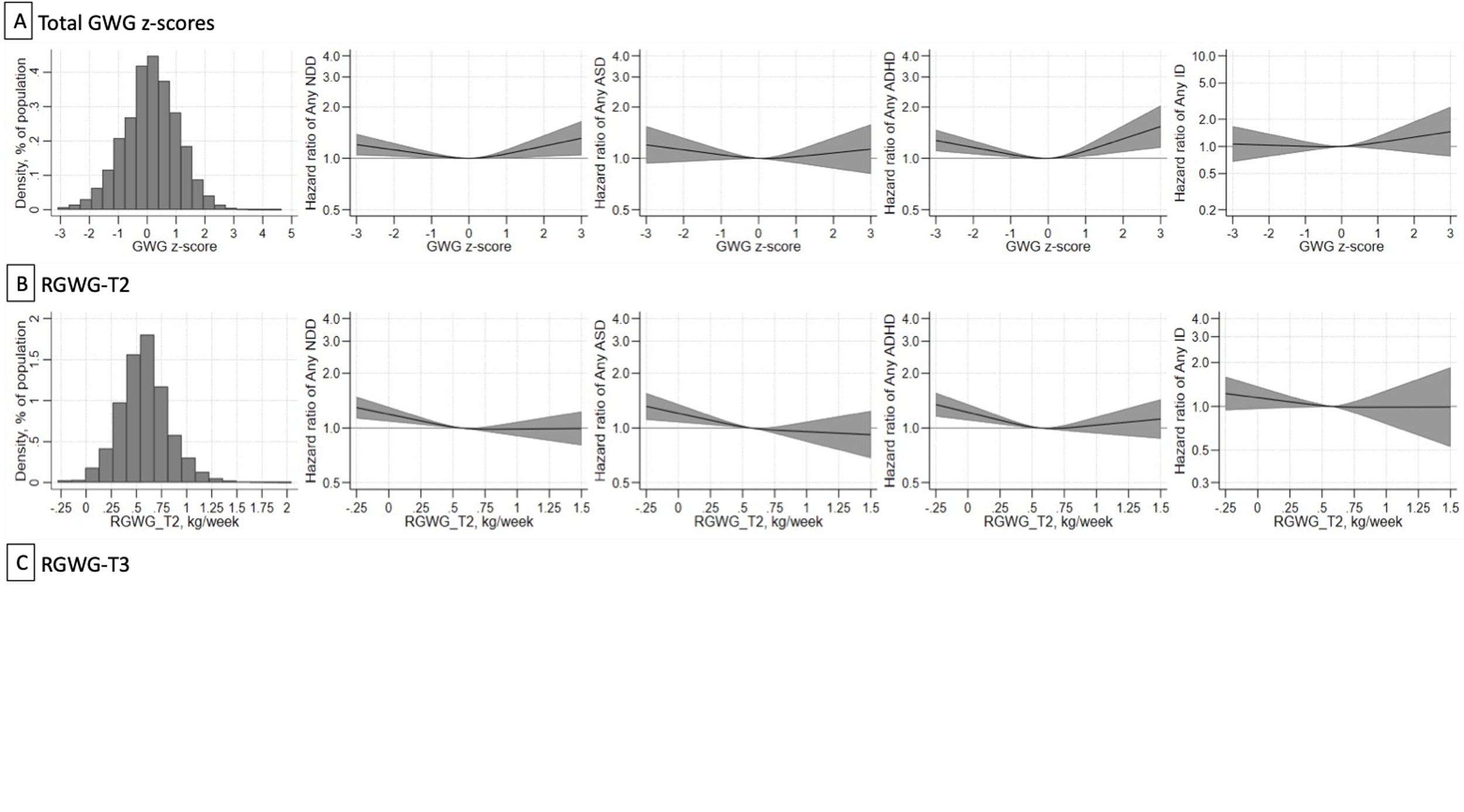
**Fig S9. Offspring risk for neurodevelopment disorders with respect to rate of gestational weight gain during second trimester (RGWG-T2), with additional adjustment for number of antenatal visits in the second trimester, with last weight measured < 25 and ≥ 25 weeks of gestation.** Histograms illustrate the distribution of RGWG-T2 for those included in each analysis. Adjusted estimates are shown for any NDDs, ASD, ADHD, and ID. The curved solid black line represents the hazard ratio (HR) calculated through restricted cubic splines models with 3 knots. The grey bands represent the 95% CI. A reference line is included for an HR of 1.00. The model was adjusted for birth year, sex, maternal age, household income quintile, maternal education level, parental birth region, interpregnancy interval, maternal psychiatric history, maternal smoking during pregnancy, and maternal BMI at first antenatal visit. In Fig E, we further adjusted for GDM and pre-eclampsia as these conditions usually occurs in late second trimester which could be potential confounders in the associations.


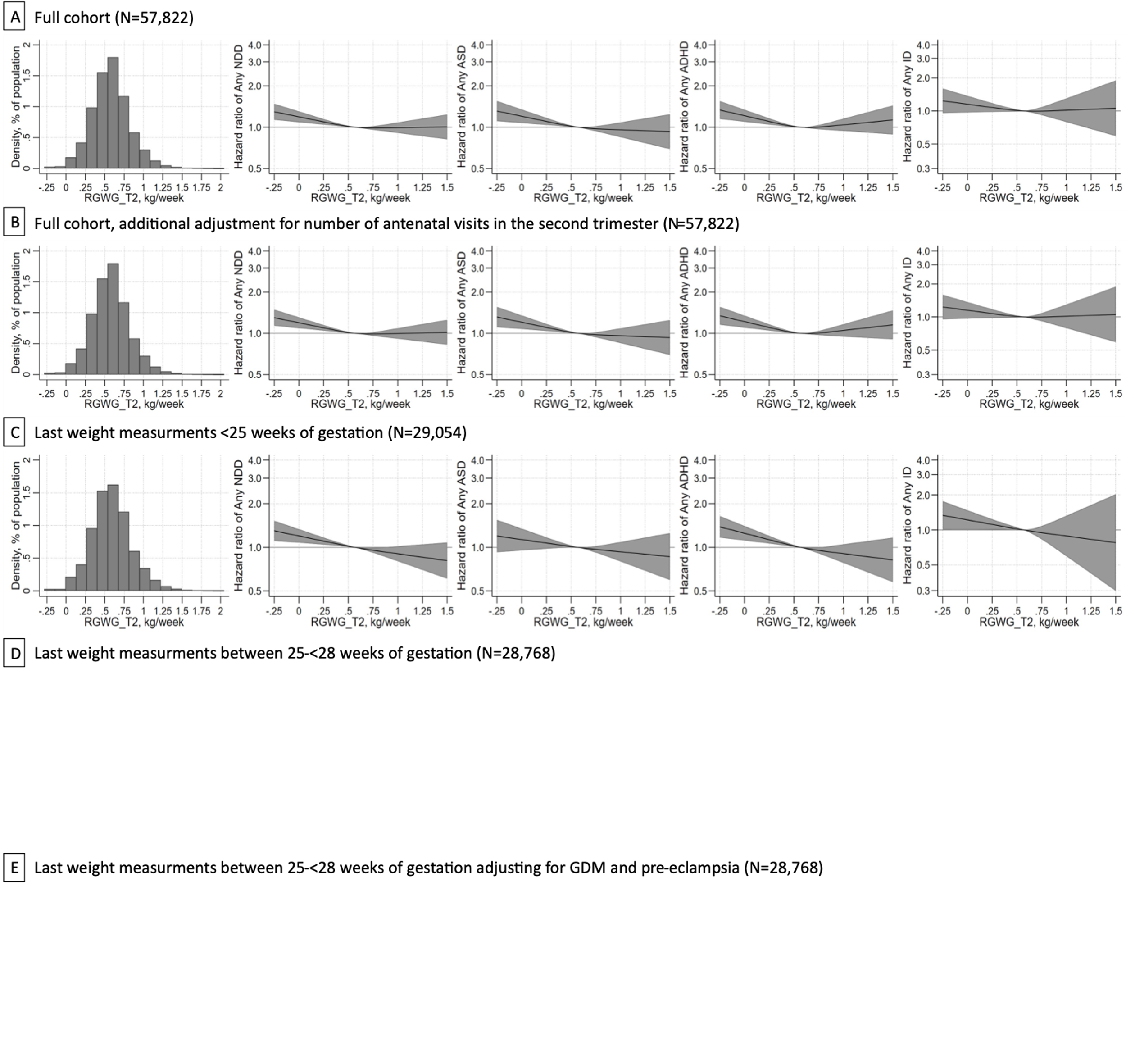


**Fig S10. The potential impact of selection bias in the association of total GWG z-scores, RGWG-T2, and RGWG-T3 with NDDs in offspring.** Histograms illustrate the distribution of GWG z-scores, RGWG-T2, and -T3 for those included in each analysis. Inverse probability weights (IPW) were applied to correct the analysis by weighting the observations with the probability of being selected. Adjusted estimates are shown for any NDDs, ASD, ADHD, and ID. The curved solid black line represents the Hazard ratio (HR) calculated through restricted cubic splines models with 3 knots with weights applied and the grey bands represent the 95% CI. The curved red dash line represents the Hazard ratio (HR) calculated through analyses without weights applied. A reference line is included for an HR of 1.00. The model was adjusted for birth year, sex, maternal age, household income quintile, maternal education level, parental birth region, interpregnancy interval, maternal psychiatric history, maternal smoking during pregnancy, and maternal BMI at first antenatal visit. Note that the y-scale differs for ID compared to the other outcomes.

**
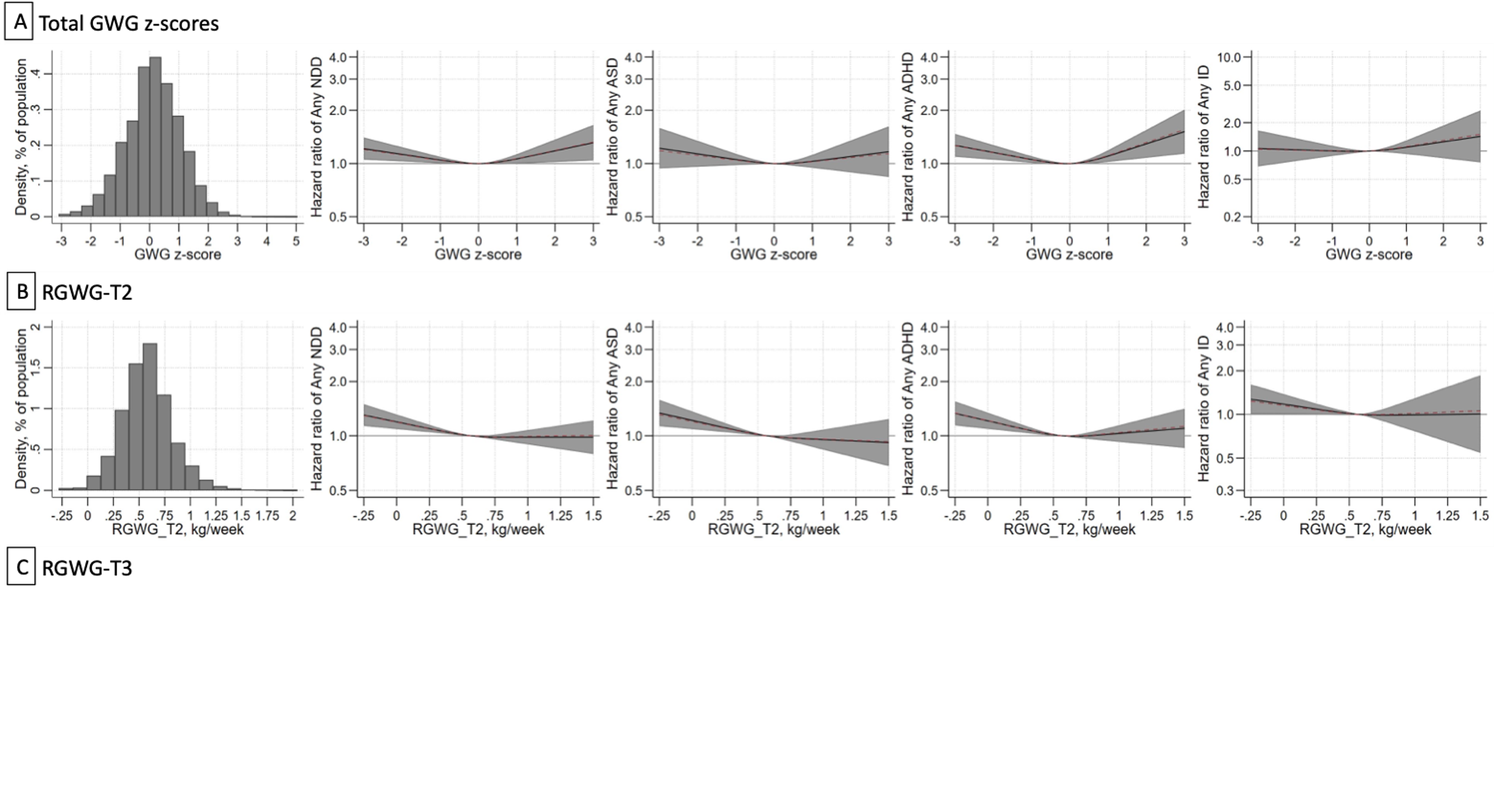
**
