# Supplementary material for: Rates of maternal weight gain over the course of pregnancy and offspring risk of neurodevelopmental disorders
Source: BMC Med. 2023 Mar 23;21:108. doi: 10.1186/s12916-023-02799-6 (PMC10035205; doi:10.1186/s12916-023-02799-6)
Supplement: Supplementary file 2 — Additional file 2. STROBE checklist. The STROBE checklist showing our study was reported according to the STROBE checklist for cohort studies. [file 12916_2023_2799_MOESM2_ESM.doc]

STROBE Statement—Checklist of items that should be included in reports of ***cohort studies***

|  | Item No | Recommendation | Location |
| --- | --- | --- | --- |
| **Title and abstract** | 1 | (*a*) Indicate the study’s design with a commonly used term in the title or the abstract | Title (p1) |
|  |  | (*b*) Provide in the abstract an informative and balanced summary of what was done and what was found | Abstract (p3-4) |
| Introduction | | |  |
| Background/rationale | 2 | Explain the scientific background and rationale for the investigation being reported | Introduction: paragraphs 2 to 3 (p5-6) |
| Objectives | 3 | State specific objectives, including any prespecified hypotheses | Introduction: paragraph 4  (p6) |
| Methods | | |  |
| Study design | 4 | Present key elements of study design early in the paper | Methods: Study population (p6) |
| Setting | 5 | Describe the setting, locations, and relevant dates, including periods of recruitment, exposure, follow-up, and data collection | Methods: Study population (p6) |
| Participants | 6 | (*a*) Give the eligibility criteria, and the sources and methods of selection of participants | Methods: Study population (p6), and FigA S1 |
| Variables | 7 | Clearly define all outcomes, exposures, predictors, potential confounders, and effect modifiers. Give diagnostic criteria, if applicable | Methods: Case Ascertainment, Exposures, Covariates (p7-9), FigB S1, Table S1 |
| Data sources/ measurement | 8* | For each variable of interest, give sources of data and details of methods of assessment (measurement). Describe comparability of assessment methods if there is more than one group | Methods: Case Ascertainment, Exposures, Covariates (p7-9), S1 Table |
| Bias | 9 | Describe any efforts to address potential sources of bias | Methods: Statistical analysis- (p11); Results (p 16, line 382-384); Table S2; Discussion- strengths and limitations (p22-23) |
| Study size | 10 | Explain how the study size was arrived at | Methods: Study population (p6-7); Results: Study sample (p12); Fig A S1 |
| Quantitative variables | 11 | Explain how quantitative variables were handled in the analyses. If applicable, describe which groupings were chosen and why | Methods: Case ascertainment, Exposures, Covariates (p7-9); Table 1, Table S3; FigB S1 |
| Statistical methods | 12 | (*a*) Describe all statistical methods, including those used to control for confounding | Methods: Statistical analysis-paragraph 1 (p9-10) |
|  |  | (*b*) Describe any methods used to examine subgroups and interactions | Methods: Statistical analysis-paragraph 2 (p10-11) |
|  |  | (*c*) Explain how missing data were addressed | Methods: Statistical analysis- paragraph 1 (p10) |
|  |  | (*d*) If applicable, describe analytical methods taking account of sampling strategy | Not applicable |
|  |  | (*e*) Describe any sensitivity analyses | Methods: Statistical analysis (p11) |
| Results | | |  |
| Participants | 13* | (a) Report numbers of individuals at each stage of study—eg numbers potentially eligible, examined for eligibility, confirmed eligible, included in the study, completing follow-up, and analysed | Methods: Study population (p6); FigA S1; Results, Study sample (p12) |
|  |  | (b) Give reasons for non-participation at each stage | Not Applicable |
|  |  | (c) Consider use of a flow diagram | FigA S1 |
| Descriptive data | 14* | (a) Give characteristics of study participants (eg demographic, clinical, social) and information on exposures and potential confounders | Results: Study Sample (p12); Table 1; Table S3, Fig 1 |
|  |  | (b) Indicate number of participants with missing data for each variable of interest | Results: Table 1; Table S3 |
| Outcome data | 15* | Report numbers of outcome events or summary measures | Results: Study sample (p12); FigB S1 |
| Main results | 16 | (*a*) Give unadjusted estimates and, if applicable, confounder-adjusted estimates and their precision (eg, 95% confidence interval). Make clear which confounders were adjusted for and why they were included | Results: Fig 2, Fig 3, Fig 4, Table 2; Table S4-6 |
|  |  | (*b*) Report category boundaries when continuous variables were categorized | Results: Table 2; Table S3 |
|  |  | (*c*) If relevant, consider translating estimates of relative risk into absolute risk for a meaningful time period | Hazard ratios reported throughout the study |
| Other analyses | 17 | Report other analyses done—eg analyses of subgroups and interactions, and sensitivity analyses | Results: Sensitivity analyses (p15-16); Fig S5-10 |
| Discussion | | |  |
| Key results | 18 | Summarise key results with reference to study objectives | Discussion: paragraph 1 (p16) |
| Limitations | 19 | Discuss limitations of the study, taking into account sources of potential bias or imprecision. Discuss both direction and magnitude of any potential bias | Discussion: Strengths and limitations- paragraph 2 (p22-23) |
| Interpretation | 20 | Give a cautious overall interpretation of results considering objectives, limitations, multiplicity of analyses, results from similar studies, and other relevant evidence | Discussion: Comparison with previous studies (p17-19); Potential mechanisms (p19-21) |
| Generalisability | 21 | Discuss the generalisability (external validity) of the study results | Discussion: Strengths and limitations-paragraph 2 (p23) |
| Other information | | |  |
| Funding | 22 | Give the source of funding and the role of the funders for the present study and, if applicable, for the original study on which the present article is based | Declaration section |

*Give information separately for exposed and unexposed groups.

**Note:** An Explanation and Elaboration article discusses each checklist item and gives methodological background and published examples of transparent reporting.
